# Association of Socioeconomic Characteristics With Disparities in COVID-19 Outcomes in Japan

**DOI:** 10.1001/jamanetworkopen.2021.17060

**Published:** 2021-07-14

**Authors:** Yuki Yoshikawa, Ichiro Kawachi

**Affiliations:** 1 Department of Social and Behavioral Sciences, Harvard T.H. Chan School of Public Health, Boston, Massachusetts

## Abstract

**Importance:**

Socioeconomic factors in the disparities in COVID-19 outcomes have been reported in studies from the US and other Western countries. However, no studies have documented national- or subnational-level outcome disparities in Asian countries.

**Objective:**

To assess the association between regional COVID-19 outcome disparities and socioeconomic characteristics in Japan.

**Design, Setting, and Participants:**

This cross-sectional study collected and analyzed confirmed COVID-19 cases and deaths (through February 13, 2021) as well as population and socioeconomic data in all 47 prefectures in Japan. The data sources were government surveys for which prefecture-level data were available.

**Exposures:**

Prefectural socioeconomic characteristics included mean annual household income, Gini coefficient, proportion of the population receiving public assistance, educational attainment, unemployment rate, employment in industries with frequent close contacts with the public, household crowding, smoking rate, and obesity rate.

**Main Outcomes and Measures:**

Rate ratios (RRs) of COVID-19 incidence and mortality by prefecture-level socioeconomic characteristics.

**Results:**

All 47 prefectures in Japan (with a total population of 126.2 million) were included in this analysis. A total of 412 126 confirmed COVID-19 cases (326.7 per 100 000 people) and 6910 deaths (5.5 per 100 000 people) were reported as of February 13, 2021. Elevated adjusted incidence and mortality RRs of COVID-19 were observed in prefectures with the lowest household income (incidence RR: 1.45 [95% CI, 1.43-1.48] and mortality RR: 1.81 [95% CI, 1.59-2.07]); highest proportion of the population receiving public assistance (1.55 [95% CI, 1.52-1.58] and 1.51 [95% CI, 1.35-1.69]); highest unemployment rate (1.56 [95% CI, 1.53-1.59] and 1.85 [95% CI, 1.65-2.09]); highest percentage of workers in retail industry (1.36 [95% CI, 1.34-1.38] and 1.45 [95% CI, 1.31-1.61]), transportation and postal industries (1.61 [95% CI, 1.57-1.64] and 2.55 [95% CI, 2.21-2.94]), and restaurant industry (2.61 [95% CI, 2.54-2.68] and 4.17 [95% CI, 3.48-5.03]); most household crowding (1.35 [95% CI, 1.31-1.38] and 1.04 [95% CI, 0.87-1.24]); highest smoking rate (1.63 [95% CI, 1.60-1.66] and 1.54 [95% CI, 1.33-1.78]); and highest obesity rate (0.93 [95% CI, 0.91-0.95] and 1.17 [95% CI, 1.01-1.34]) compared with prefectures with the most social advantages. Among potential mediating variables, higher smoking rate (RR, 1.54; 95% CI, 1.33-1.78) and obesity rate (RR, 1.17; 95% CI, 1.01-1.34) were associated with higher mortality RRs, even after adjusting for prefecture-level covariates and other socioeconomic variables.

**Conclusions and Relevance:**

This cross-sectional study found a pattern of socioeconomic disparities in COVID-19 outcomes in Japan that was similar to that observed in the US and Europe. National policy in Japan could consider prioritizing populations in socially disadvantaged regions in the COVID-19 response, such as vaccination planning, to address this pattern.

## Key Points

**Question:**

Are the COVID-19 outcome disparities between Japanese regions associated with the socioeconomic characteristics of those regions?

**Findings:**

In this cross-sectional study of the 47 prefectures in Japan, a higher burden of COVID-19 cases and deaths was observed in prefectures with lower household incomes; a higher proportion of the population receiving public assistance; a higher unemployment rate; higher numbers of retail, transportation and postal, and restaurant industry workers; more household crowding; and higher smoking and obesity rates.

**Meaning:**

This study found an unequal pattern of COVID-19 outcomes that was associated with the socioeconomic circumstances in Japanese regions, suggesting that these disparities in COVID-19 outcomes are not unique to the US and Europe.

## Teaser

This cross-sectional study examines the association of socioeconomic characteristics with COVID-19 incidence and mortality in Japanese prefectures.

## Introduction

Since the emergence of SARS-CoV-2 in 2019, more than 100 million cases of COVID-19 and 2.3 million deaths from COVID-19 have been reported worldwide as of February 14, 2021.^1^ Although older age has been shown to be a major risk factor of COVID-19 mortality,^2^ several US studies have suggested that morbidity and mortality from this disease are associated with socioeconomic circumstances, including income, educational attainment, employment in service or retail industry, area poverty level, unemployment, household crowding, and race/ethnicity.^3,4,5,6^

Understanding the sources of vulnerability to COVID-19 is essential for planning and delivery of interventions, such as deciding which individuals or groups should receive priority for the vaccine. Although most studies of COVID-19 outcome disparities have originated from the US, studies from other countries, including the UK, Italy, Spain, Brazil, Mexico, and Ecuador, have found similar patterns.^7,8,9,10,11,12^ However, thus far, only 1 cross-national study has evaluated the socioeconomic factors in the disparities in COVID-19 outcomes among Asian countries,^13^ and no research has focused on national or subnational disparities.

Japan reported the first case of COVID-19 on January 16, 2020.^14^ In Japan, local public health centers play a pivotal role in the pandemic response, including triaging, testing, allocating individuals to hospitals, and contact tracing. The Japanese government also covers all COVID-19–related medical costs, including public testing and hospital care, although people still need to pay out-of-pocket fees for nonpublic (ie, commercially available) tests. Japan is divided into 47 prefectures, which are the first-level administrative divisions. Population size varies widely between prefectures; of the total population of 126.2 million people, 13.9 million live in Tokyo, the most populous prefecture, and 0.6 million people live in Tottori, the least populous prefecture.^15^ The most densely populated, urban prefectures are situated along the Pacific coast between Tokyo, Aichi, and Osaka. The northeastern prefectures (Tohoku region), the prefectures facing the Japan Sea, and the southern island of Shikoku are generally more rural, less densely populated, and inhabited by older adults. During the first wave of the pandemic, clusters of COVID-19 cases were reported in the Tokyo, Aichi, and Osaka metropolitan areas as well as the northern island of Hokkaido (which is its own prefecture). The first outbreak in Hokkaido was believed to be linked to travelers from China.^16^

Case and death data show a wide variability in the burden of COVID-19 between prefectures,^17^ and the reasons for this variability are not fully understood. We hypothesized that this variability is at least partly associated with socioeconomic disparities by region. Accordingly, we conducted a cross-sectional study to assess the association between regional COVID-19 outcome disparities and socioeconomic characteristics in Japan, including income and wealth, educational attainment, occupation and unemployment, living conditions, and health-related factors.

## Methods

### Study Design

For this cross-sectional prefecture-level ecological study, we extracted publicly available COVID-19 and socioeconomic data from government sources. Given that the most granular data were available at the prefectural level, the association of COVID-19 outcome disparities with socioeconomic characteristics was evaluated across all 47 prefectures in Japan. We followed the Strengthening the Reporting of Observational Studies in Epidemiology (STROBE) reporting guideline. This study was considered exempt from institutional review board review and required no informed consent because it used deidentified, publicly available data.

### Data Sources

In this study, the cumulative number of confirmed COVID-19 cases and deaths were designated as the burden of disease. The Ministry of Health, Labour and Welfare of Japan updates daily the data on the cumulative number of cases with positive COVID-19 test results and deaths at the prefectural and national levels.^17^ Because the accumulated number of hospitalized cases has not been reported and the COVID-19 admission criteria are different by region and timing within the pandemic, hospital admission data were not used in the current study. To analyze the health outcomes at the prefectural level, we excluded cases and deaths that were not linked to any prefectures from the analysis. Then, we merged COVID-19 outcome data with population data by prefecture. The population data are annually updated by the Ministry of Internal Affairs and Communications of Japan, and we used the most recent data available (October 1, 2019).^15^ Because the Japanese government has not reported prefecture-level COVID-19 outcomes disaggregated by age or sex, we used the crude outcome rates instead of standardized rates.

We collected socioeconomic data from multiple government surveys. The specific socioeconomic variables analyzed in this study were as follows: mean annual household income (adjusted by regional price parities),^18,19^ Gini coefficient (for income inequality),^18^ proportion of the population receiving public assistance (cash assistance and in-kind benefit program for poor households),^20^ educational attainment (percentage of graduates aged 20 years or older with a college or a higher-level degree),^21^ unemployment rate,^22^ employment in industries with frequent close contacts with the public (percentage of workers in the health care, retail, transportation and postal, and restaurant industries),^21^ household crowding (living area per person; lower values were associated with more crowding),^23^ smoking rate,^24^ and obesity rate^25^ (eTables 1 and 2 in the Supplement). The data were extracted from the latest government surveys for which prefecture-level data were available. Each variable was divided into quintiles at quintile cutoff values (eTable 3 in the Supplement). The number of cases and deaths per 100 000 residents were calculated for each quintile of the variables.

Among the prefecture-level covariates for which we adjusted were percentage of the older adult population,^15^ population density,^26^ and number of acute care hospital beds per population.^27^ Data showed that, in Japan, the number of COVID-19 deaths was higher among the older adult population, whereas COVID-19 incidence was higher among younger people.^17^ Acute care hospital beds were considered a proxy for health care access, as it is a measure of local capacity to conduct testing as well as provide acute care for residents.

### Statistical Analysis

We used Poisson regression to evaluate the association between COVID-19 outcomes and socioeconomic characteristics across 47 prefectures. The logarithms of the number of cases and deaths at the prefecture level were set as the outcome variable, with the logarithm of the prefectural population included as an offset term. Specifically, we estimated the coefficients of the following model using Poisson regression:

log(y_i_) = α + β × SEC_i_ + γ × x_i_ + log(population_i_) + ε_i_,

where y_i_ was the outcome (COVID-19 cases or deaths) for prefecture i; SEC_i_ was a vector of socioeconomic characteristics for the prefecture i; x_i_ was a vector of prefecture-level covariates, including the percentage of the older adult population, population density, and number of acute care hospital beds per population; and population_i_ was the population of each prefecture. As a measure of relative disparities, we reported the incidence and mortality rate ratios (RRs), exponentiated coefficients of the Poisson regression model, with 95% CIs by designating the group with the most social advantages as the reference group, excluding the workers in specific industries, for whom the group with the lowest percentage employed in a specific industry was set as the reference group.

We investigated the bivariate associations between COVID-19 outcomes and each of the socioeconomic characteristics, a process we termed model 1. Next, we adjusted model 1 for the 3 prefecture-level characteristics (percentage of the older adult population, population density, and number of acute care hospital beds per population), a process we termed model 2. For variables that we considered to be potential mediating variables of an association between socioeconomic circumstances and COVID-19 outcomes (household crowding, smoking rate, and obesity rate), we further adjusted model 2 for household income adjusted by regional price parities, Gini coefficient, the proportion of the population receiving public assistance, educational attainment, and unemployment rate, a process we termed model 3. This analysis aimed to check whether the coefficients between the potential mediating variables and COVID-19 outcomes were attenuated by including other socioeconomic variables; if attenuated, the observed association in model 2 was confounded by other socioeconomic variables. In addition, we performed a mediation analysis to investigate whether the potential mediating variables moderated the association between socioeconomic circumstances and COVID-19 outcomes. We checked whether the exponentiated coefficients between the association became attenuated after incorporating each variable (household crowding, smoking rate, or obesity rate) into model 2.

We conducted 2 sensitivity analyses to assess the robustness of the regression model. In the first sensitivity analysis, we incorporated the number of polymerase chain reaction (PCR) tests per population in each prefecture into models 2 and 3.^17^ The number included only the PCR tests that were reported to the local or national government; thus, it did not necessarily represent all of the COVID-19 tests conducted. However, the number can be used as a proxy for test accessibility in each prefecture. In the second sensitivity analysis, following Chen and Krieger,^4^ we assessed relative disparities on the basis of indirect standardization to account for the possible differences in sex and age distribution by prefecture. We calculated the national age- and sex-specific COVID-19 incidence and mortality rates and multiplied these rates by the prefectural age- and sex-specific population.^15,17^ By summing the cases and deaths, we calculated the expected cases and deaths for each prefecture. These values were used as an offset term for models 1, 2, and 3 instead of the prefectural population.

All analyses were performed using R, version 4.0.3 (R Foundation for Statistical Computing).

## Results

A total of 412 275 confirmed COVID-19 cases and 6910 deaths in Japan were reported as of February 13, 2021. Of the total cases, 149 individuals were excluded from analysis because they were cruise ship passengers and not linked to any prefectures in the government report. In 1 prefecture (Shimane), COVID-19 deaths have not been reported yet. The number of cases per 100 000 people was 326.7, and the number of deaths per 100 000 people was 5.5 (eTable 4 in the Supplement).

As shown in the Table, in model 1, we observed higher incidence RRs and mortality RRs in prefectures with the most socioeconomic disadvantages in terms of Gini coefficient (incidence RR: 2.63 [95% CI, 2.60-2.66] and mortality RR: 2.36 [95% CI, 2.17-2.57]), the proportion of the population receiving public assistance (2.45 [95% CI, 2.43-2.48] and 2.02 [95% CI, 1.88-2.18]), unemployment rate (2.60 [95% CI, 2.56-2.65] and 2.58 [95% CI, 2.31-2.89]), percentage of workers in transportation and postal industries (3.37 [95% CI, 3.31-3.43] and 3.33 [95% CI, 2.92-3.82]) and restaurant industry (6.51 [95% CI, 6.37-6.66] and 4.84 [95% CI, 4.14-5.71]), household crowding (5.28 [95% CI, 5.19-5.38] and 3.27 [95% CI, 2.92-3.68]), and obesity rate (1.04 [95% CI, 1.03-1.05] and 1.23 [95% CI, 1.12-1.35]).

**Table.** Japanese COVID-19 Incidence Rate, Mortality Rate, Incidence Rate Ratio, and Mortality Rate Ratio by Prefectural Socioeconomic Characteristics as of February 13, 2021

Socioeconomic variable	Incidence rate per 100 000 (95% CI)	Incidence RR (95% CI)^a^			Mortality rate per 100 000 (95% CI)	Mortality RR (95% CI)^a^		
		Model 1^b^	Model 2^c^	Model 3^d^		Model 1^b^	Model 2^c^	Model 3^d^
Household income adjusted by regional price parities								
5th quintile	249.7 (247.9-251.6)	1 [Reference]	1 [Reference]	NA	4.35 (4.11-4.60)	1 [Reference]	1 [Reference]	NA
4th quintile	463.7 (461.7-465.8)	1.86 (1.84-1.87)	0.99 (0.98-1.00)	NA	5.94 (5.71-6.18)	1.37 (1.28-1.46)	0.78 (0.71-0.85)	NA
3rd quintile	174.3 (172.3-176.4)	0.70 (0.69-0.71)	1.02 (1.00-1.03)	NA	4.00 (3.69-4.33)	0.92 (0.84-1.01)	1.17 (1.05-1.30)	NA
2nd quintile	335.5 (333.2-337.8)	1.34 (1.33-1.36)	1.31 (1.30-1.33)	NA	6.68 (6.37-7.01)	1.54 (1.43-1.66)	1.34 (1.22-1.49)	NA
1st quintile	235.3 (232.8-237.7)	0.94 (0.93-0.95)	1.45 (1.43-1.48)	NA	5.86 (5.48-6.26)	1.35 (1.24-1.47)	1.81 (1.59-2.07)	NA
Gini coefficient								
1st quintile	193.8 (192.0-195.6)	1 [Reference]	1 [Reference]	NA	3.07 (2.84-3.31)	1 [Reference]	1 [Reference]	NA
2nd quintile	206.4 (204.4-208.4)	1.07 (1.05-1.08)	1.05 (1.04-1.07)	NA	4.65 (4.35-4.96)	1.51 (1.37-1.67)	1.32 (1.19-1.47)	NA
3rd quintile	335.1 (333.1-337.1)	1.73 (1.71-1.75)	0.96 (0.95-0.97)	NA	5.50 (5.24-5.76)	1.79 (1.64-1.96)	1.25 (1.13-1.37)	NA
4th quintile	270.7 (268.4-273.1)	1.40 (1.38-1.42)	1.34 (1.32-1.37)	NA	6.04 (5.70-6.39)	1.97 (1.79-2.16)	1.38 (1.22-1.56)	NA
5th quintile	510.0 (507.5-512.4)	2.63 (2.60-2.66)	0.81 (0.80-0.83)	NA	7.23 (6.94-7.53)	2.36 (2.17-2.57)	0.79 (0.69-0.90)	NA
Proportion of the population receiving public assistance								
1st quintile	206.1 (204.2-208.1)	1 [Reference]	1 [Reference]	NA	4.05 (3.78-4.33)	1 [Reference]	1 [Reference]	NA
2nd quintile	183.9 (182.0-185.7)	0.89 (0.88-0.90)	1.11 (1.10-1.13)	NA	4.21 (3.94-4.51)	1.04 (0.95-1.15)	1.11 (1.01-1.22)	NA
3rd quintile	138.7 (136.7-140.7)	0.67 (0.66-0.68)	0.92 (0.91-0.94)	NA	2.28 (2.03-2.54)	0.56 (0.49-0.64)	0.63 (0.55-0.72)	NA
4th quintile	354.2 (352.1-356.3)	1.72 (1.70-1.74)	1.55 (1.54-1.57)	NA	5.22 (4.97-5.48)	1.29 (1.19-1.40)	1.23 (1.13-1.34)	NA
5th quintile	505.7 (503.4-507.9)	2.45 (2.43-2.48)	1.55 (1.52-1.58)	NA	8.18 (7.90-8.47)	2.02 (1.88-2.18)	1.51 (1.35-1.69)	NA
Educational attainment: college or higher-level degree								
5th quintile	470.0 (468.3-471.6)	1 [Reference]	1 [Reference]	NA	7.37 (7.16-7.58)	1 [Reference]	1 [Reference]	NA
4th quintile	183.5 (181.6-185.4)	0.39 (0.39-0.39)	0.58 (0.57-0.58)	NA	2.86 (2.63-3.11)	0.39 (0.36-0.42)	0.38 (0.34-0.42)	NA
3rd quintile	199.5 (197.3-201.7)	0.42 (0.42-0.43)	0.70 (0.69-0.71)	NA	3.47 (3.19-3.77)	0.47 (0.43-0.51)	0.54 (0.49-0.60)	NA
2nd quintile	213.6 (211.1-216.1)	0.45 (0.45-0.46)	0.78 (0.77-0.80)	NA	5.90 (5.49-6.32)	0.80 (0.74-0.86)	0.66 (0.59-0.74)	NA
1st quintile	80.7 (79.1-82.2)	0.17 (0.17-0.18)	0.37 (0.37-0.38)	NA	1.72 (1.50-1.97)	0.23 (0.20-0.27)	0.26 (0.22-0.30)	NA
Unemployment rate								
1st quintile	127.8 (125.8-129.8)	1 [Reference]	1 [Reference]	NA	2.79 (2.50-3.09)	1 [Reference]	1 [Reference]	NA
2nd quintile	198.4 (196.6-200.3)	1.55 (1.52-1.58)	1.03 (1.01-1.05)	NA	3.54 (3.30-3.80)	1.27 (1.12-1.45)	1.08 (0.95-1.23)	NA
3rd quintile	318.6 (316.4-320.9)	2.49 (2.45-2.54)	1.41 (1.38-1.44)	NA	4.74 (4.47-5.02)	1.70 (1.51-1.92)	1.31 (1.15-1.49)	NA
4th quintile	471.0 (468.8-473.2)	3.69 (3.63-3.75)	1.40 (1.38-1.43)	NA	6.57 (6.31-6.84)	2.36 (2.11-2.64)	1.38 (1.22-1.57)	NA
5th quintile	332.8 (330.8-334.9)	2.60 (2.56-2.65)	1.56 (1.53-1.59)	NA	7.19 (6.89-7.49)	2.58 (2.31-2.89)	1.85 (1.65-2.09)	NA
Percentage of workers in health care industry								
1st quintile	439.4 (437.7-441.2)	1 [Reference]	1 [Reference]	NA	5.99 (5.78-6.19)	1 [Reference]	1 [Reference]	NA
2nd quintile	140.4 (138.6-142.2)	0.32 (0.32-0.32)	0.68 (0.67-0.69)	NA	2.61 (2.37-2.87)	0.44 (0.39-0.48)	0.65 (0.58-0.72)	NA
3rd quintile	333.3 (331.2-335.4)	0.76 (0.75-0.76)	1.27 (1.25-1.28)	NA	8.44 (8.11-8.78)	1.41 (1.34-1.49)	1.72 (1.58-1.87)	NA
4th quintile	235.3 (232.9-237.7)	0.54 (0.53-0.54)	0.91 (0.89-0.92)	NA	3.17 (2.89-3.46)	0.53 (0.48-0.58)	0.61 (0.54-0.68)	NA
5th quintile	122.1 (119.9-124.4)	0.28 (0.27-0.28)	0.77 (0.75-0.79)	NA	2.04 (1.77-2.35)	0.34 (0.29-0.39)	0.55 (0.46-0.65)	NA
Percentage of workers in retail industry								
1st quintile	483.6 (481.3-485.8)	1 [Reference]	1 [Reference]	NA	7.09 (6.83-7.37)	1 [Reference]	1 [Reference]	NA
2nd quintile	284.8 (282.9-286.6)	0.59 (0.58-0.59)	1.25 (1.23-1.27)	NA	4.80 (4.56-5.05)	0.68 (0.64-0.72)	1.44 (1.30-1.59)	NA
3rd quintile	314.9 (312.9-316.9)	0.65 (0.65-0.66)	1.19 (1.17-1.20)	NA	5.52 (5.25-5.79)	0.78 (0.73-0.83)	1.30 (1.20-1.41)	NA
4th quintile	118.2 (116.3-120.1)	0.24 (0.24-0.25)	0.77 (0.76-0.79)	NA	1.52 (1.32-1.76)	0.21 (0.18-0.25)	0.44 (0.37-0.51)	NA
5th quintile	222.9 (220.5-225.3)	0.46 (0.46-0.47)	1.36 (1.34-1.38)	NA	6.08 (5.70-6.49)	0.86 (0.80-0.92)	1.45 (1.31-1.61)	NA
Percentage of workers in transportation and postal industry								
1st quintile	114.4 (112.3-116.4)	1 [Reference]	1 [Reference]	NA	2.14 (1.87-2.44)	1 [Reference]	1 [Reference]	NA
2nd quintile	224.4 (221.9-226.9)	1.96 (1.92-2.00)	1.27 (1.24-1.30)	NA	3.72 (3.40-4.05)	1.73 (1.49-2.03)	1.38 (1.17-1.63)	NA
3rd quintile	108.5 (106.7-110.3)	0.95 (0.93-0.97)	0.78 (0.76-0.80)	NA	2.05 (1.82-2.31)	0.96 (0.80-1.14)	0.90 (0.76-1.08)	NA
4th quintile	423.8 (421.6-426.0)	3.71 (3.64-3.77)	1.29 (1.26-1.32)	NA	5.88 (5.62-6.14)	2.74 (2.40-3.15)	1.78 (1.54-2.08)	NA
5th quintile	385.3 (383.7-386.9)	3.37 (3.31-3.43)	1.61 (1.57-1.64)	NA	7.14 (6.91-7.36)	3.33 (2.92-3.82)	2.55 (2.21-2.94)	NA
Percentage of workers in restaurant industry								
1st quintile	76.3 (74.6-78.0)	1 [Reference]	1 [Reference]	NA	1.53 (1.30-1.79)	1 [Reference]	1 [Reference]	NA
2nd quintile	120.7 (119.0-122.4)	1.58 (1.54-1.62)	1.44 (1.40-1.48)	NA	2.42 (2.18-2.68)	1.58 (1.31-1.91)	1.56 (1.30-1.89)	NA
3rd quintile	151.7 (149.7-153.7)	1.99 (1.94-2.04)	1.55 (1.51-1.60)	NA	2.33 (2.08-2.59)	1.52 (1.26-1.84)	1.56 (1.29-1.90)	NA
4th quintile	277.1 (275.2-279.0)	3.63 (3.55-3.72)	2.82 (2.75-2.88)	NA	6.23 (5.95-6.53)	4.07 (3.47-4.81)	4.19 (3.55-4.98)	NA
5th quintile	496.6 (494.7-498.4)	6.51 (6.37-6.66)	2.61 (2.54-2.68)	NA	7.42 (7.20-7.65)	4.84 (4.14-5.71)	4.17 (3.48-5.03)	NA
Household crowding								
5th quintile	95.4 (93.8-97.1)	1 [Reference]	1 [Reference]	1 [Reference]	2.23 (1.99-2.49)	1 [Reference]	1 [Reference]	1 [Reference]
4th quintile	205.1 (202.8-207.4)	2.15 (2.11-2.19)	1.81 (1.77-1.84)	1.50 (1.47-1.54)	5.80 (5.42-6.20)	2.60 (2.29-2.97)	2.16 (1.89-2.47)	2.21 (1.92-2.55)
3rd quintile	149.4 (147.5-151.4)	1.57 (1.53-1.60)	1.29 (1.27-1.32)	1.22 (1.19-1.25)	2.79 (2.53-3.06)	1.25 (1.08-1.45)	1.06 (0.91-1.23)	0.99 (0.85-1.15)
2nd quintile	287.6 (285.8-289.5)	3.01 (2.96-3.07)	1.99 (1.95-2.03)	1.65 (1.61-1.68)	5.17 (4.92-5.42)	2.32 (2.06-2.62)	2.04 (1.80-2.33)	1.62 (1.41-1.87)
1st quintile	504.2 (502.3-506.2)	5.28 (5.19-5.38)	2.04 (2.00-2.08)	1.35 (1.31-1.38)	7.30 (7.06-7.53)	3.27 (2.92-3.68)	1.84 (1.60-2.11)	1.04 (0.87-1.24)
Smoking rate								
1st quintile	476.2 (473.7-478.7)	1 [Reference]	1 [Reference]	1 [Reference]	6.43 (6.14-6.72)	1 [Reference]	1 [Reference]	1 [Reference]
2nd quintile	275.4 (273.2-277.5)	0.58 (0.57-0.58)	1.01 (1.00-1.02)	1.26 (1.24-1.28)	4.27 (4.01-4.55)	0.66 (0.61-0.72)	1.05 (0.96-1.15)	1.05 (0.94-1.17)
3rd quintile	246.8 (244.9-248.7)	0.52 (0.51-0.52)	1.00 (0.99-1.01)	1.79 (1.75-1.83)	4.47 (4.22-4.73)	0.70 (0.65-0.75)	1.32 (1.20-1.45)	1.98 (1.71-2.30)
4th quintile	336.8 (334.4-339.2)	0.71 (0.70-0.71)	1.19 (1.17-1.20)	2.05 (2.00-2.10)	6.75 (6.41-7.10)	1.05 (0.98-1.12)	1.39 (1.29-1.50)	1.65 (1.39-1.95)
5th quintile	272.1 (270.1-274.2)	0.57 (0.57-0.58)	1.38 (1.36-1.39)	1.63 (1.60-1.66)	5.41 (5.12-5.70)	0.84 (0.78-0.90)	1.45 (1.33-1.58)	1.54 (1.33-1.78)
Obesity rate								
1st quintile	205.1 (203.1-207.2)	1 [Reference]	1 [Reference]	1 [Reference]	4.45 (4.16-4.76)	1 [Reference]	1 [Reference]	1 [Reference]
2nd quintile	467.1 (465.1-469.1)	2.28 (2.25-2.30)	0.86 (0.85-0.87)	0.92 (0.90-0.93)	7.10 (6.85-7.35)	1.59 (1.48-1.72)	0.84 (0.76-0.92)	0.88 (0.78-0.99)
3rd quintile	335.1 (333.2-337.1)	1.63 (1.62-1.65)	1.12 (1.11-1.14)	0.99 (0.98-1.01)	5.05 (4.81-5.30)	1.13 (1.05-1.23)	0.97 (0.89-1.06)	0.84 (0.77-0.93)
4th quintile	148.3 (146.2-150.4)	0.72 (0.71-0.74)	0.79 (0.77-0.80)	0.91 (0.89-0.93)	2.53 (2.27-2.83)	0.57 (0.50-0.65)	0.56 (0.49-0.63)	0.71 (0.61-0.81)
5th quintile	213.3 (211.1-215.5)	1.04 (1.03-1.05)	0.89 (0.88-0.90)	0.93 (0.91-0.95)	5.47 (5.12-5.84)	1.23 (1.12-1.35)	1.03 (0.93-1.14)	1.17 (1.01-1.34)

Abbreviations: NA, not applicable; RR, rate ratio.

^a^ Incidence rate ratio and mortality rate ratio were calculated using Poisson regression models with log(population) as the offset.

^b^ Model 1 is controlled for each socioeconomic characteristic.

^c^ Model 2 is controlled for each socioeconomic characteristic and prefecture-level characteristics (percentage of the older adult population, population density, and number of acute care hospital beds per population).

^d^ Model 3 is controlled for each socioeconomic characteristic (only for household crowding, smoking rate, and obesity rate), prefecture-level characteristics (percentage of the older adult population, population density, and number of acute care hospital beds per population), and other socioeconomic characteristics (household income adjusted by regional price parities, Gini coefficient, the proportion of the population receiving public assistance, educational attainment, and unemployment rate).

**Figure 1.**
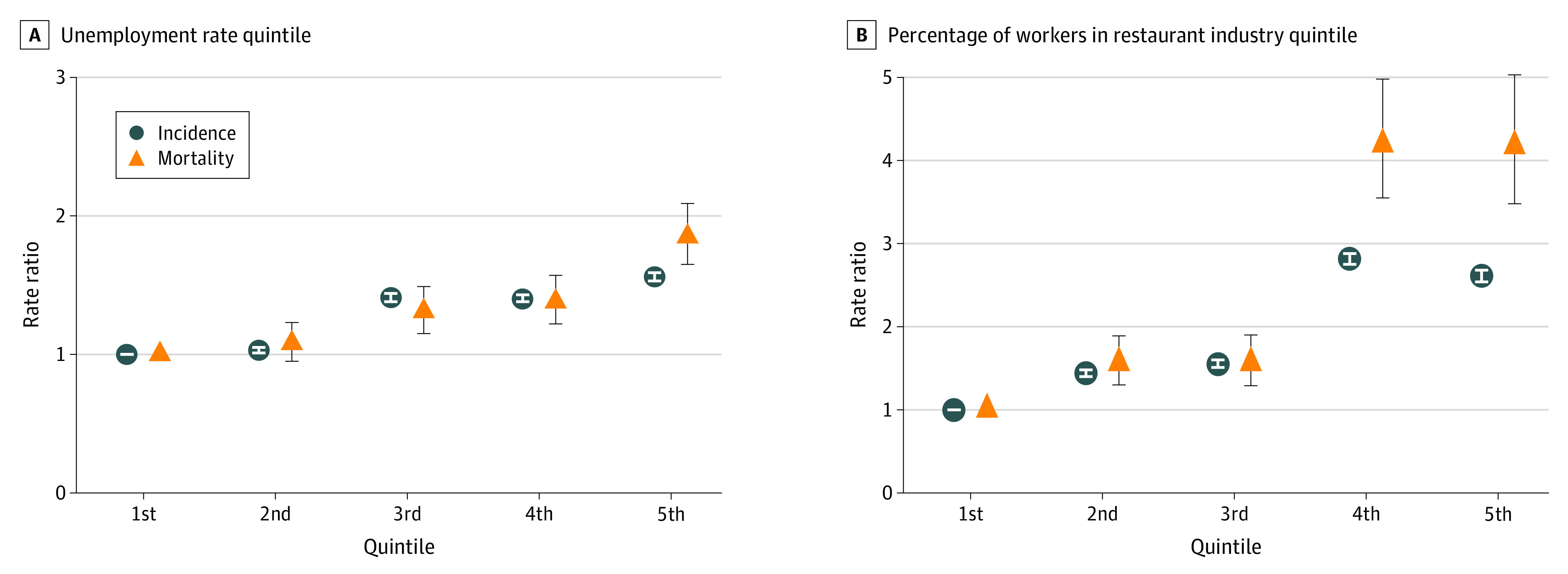
Japanese COVID-19 Incidence Rate Ratio and Mortality Rate Ratio by Prefectural Unemployment Rate Quintile and Percentage of Workers in Restaurant Industry Quintile as of February 13, 2021 Incidence and mortality rate ratios were calculated using Poisson regression models with log(population) as the offset, controlled for a socioeconomic characteristic (ie, unemployment rate or the percentage of workers in the restaurant industry) and prefecture-level characteristics (ie, percentage of the older adult population, population density, and number of acute care hospital beds per population) in model 2. Circles indicate incidence rate ratios; triangles, mortality rate ratios; error bars, 95% CIs.

Alternatively, an inverse or null association was observed for prefecture-level educational attainment (incidence RR: 0.17 [95% CI, 0.17-0.18] and mortality RR: 0.23 [95% CI, 0.20-0.27]), percentage of workers in the health care industry (0.28 [95% CI, 0.27-0.28] and 0.34 [95% CI, 0.29-0.39]) and retail industry (0.46 [95% CI, 0.46-0.47] and 0.86 [95% CI, 0.80-0.92]), and smoking rate (0.57 [95% CI, 0.57-0.58] and 0.84 [95% CI, 0.78-0.90]). These associations were mostly attenuated after adjusting for prefecture-level covariates in model 2, and the highest incidence RR (2.61; 95% CI, 2.54-2.68) and mortality RR (4.17; 95% CI, 3.48-5.03) were observed in prefectures with the highest percentage of restaurant industry workers compared with prefectures with the lowest percentage of these workers (Figure 1). In addition, monotonic socioeconomic gradients were observed for incidence RR (1.03 [95% CI, 1.01-1.05] for 2nd quintile; 1.56 [95% CI, 1.53-1.59] for 5th quintile) and mortality RR (1.08 [95% CI, 0.95-1.23] for 2nd quintile; 1.85 [95% CI, 1.65-2.09] for 5th quintile) with regard to the unemployment rate.

Adjusted higher incidence and mortality RRs were also observed in prefectures with the lowest household income (1.45 [95% CI, 1.43-1.48] and 1.81 [95% CI, 1.59-2.07]); highest proportion of the population receiving public assistance (1.55 [95% CI, 1.52-1.58] and 1.51 [95% CI, 1.35-1.69]); and highest percentage of workers in the retail industry (1.36 [95% CI, 1.34-1.38] and 1.45 [95% CI, 1.31-1.61]) and in transportation and postal industries (1.61 [95% CI, 1.57-1.64] and 2.55 [95% CI, 2.21-2.94]), compared with prefectures with the most social advantages.

**Figure 2.**
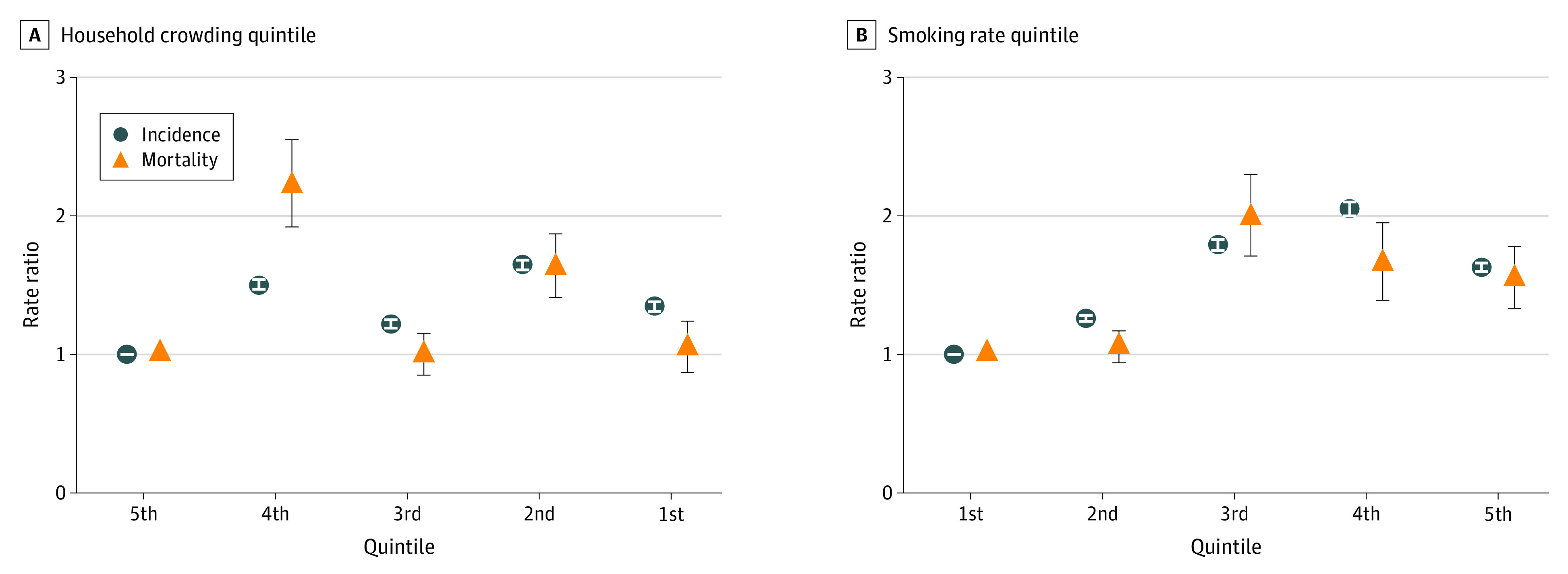
Japanese COVID-19 Incidence Rate Ratio and Mortality Rate Ratio by Prefectural Household Crowding Quintile and Smoking Rate Quintile as of February 13, 2021 Incidence and mortality rate ratios were calculated using Poisson regression models with log(population) as the offset, controlled for a socioeconomic characteristic (ie, household crowding or smoking rate), prefecture-level characteristics (ie, percentage of the older adult population, population density, and number of acute care hospital beds per population), and other socioeconomic characteristics (household income adjusted by regional price parities, Gini coefficient, proportion of the population receiving public assistance, educational attainment, and unemployment rate) in model 3. Circles indicate incidence rate ratios; triangles, mortality rate ratios; error bars, 95% CIs.

For potential mediating variables (household crowding, smoking rate, and obesity rate), we found that smaller living area per person (ie, more crowding) was associated with higher incidence rate and mortality rate in model 2, but the association disappeared for mortality RR after controlling for other socioeconomic variables in model 3 (incidence RR: 1.35 [95% CI, 1.31-1.38] and mortality RR: 1.04 [95% CI, 0.87-1.24]) (Figure 2). On the other hand, higher smoking rates (1.63 [95% CI, 1.60-1.66] and 1.54 [95% CI, 1.33-1.78]) and obesity rates (0.93 [95% CI, 0.91-0.95] and 1.17 [95% CI, 1.01-1.34]) were associated with higher mortality RRs even after adjusting for prefecture-level covariates and other socioeconomic variables. Mediation analysis showed that the association between the proportion of the population receiving public assistance and mortality RR turned null after including smoking rate among prefectures with the highest percentage of public assistance recipients (eTable 5 in the Supplement).

### Sensitivity Analysis Results

Models 2 and 3 were also adjusted for PCR tests per population to further account for test accessibility. The incidence RR and mortality RR showed similar patterns as in the main analysis (eTable 6 in the Supplement). In addition, to partially account for the sex and age differences between prefectures, we performed indirect age and sex standardization. The results of this standardization also showed patterns similar to those of the main analysis (eTable 7 in the Supplement).

## Discussion

This cross-sectional study suggests that the burden of COVID-19 was higher in socially disadvantaged regions. To our knowledge, this study was the first to investigate the association between social determinants and COVID-19 outcomes at a national level in an Asian country. Just as in Western countries, communities in regions with lower socioeconomic status were found to have greater vulnerability to COVID-19.

Previous studies from North America, South America, and Europe demonstrated that poverty level and unemployment rate were associated with the COVID-19 burden.^3,4,8,9,11^ The current study showed a similar association in Japan. Hawkins^3^ observed an association between COVID-19 cases and the proportion of the workforce engaged in providing essential services, such as in the health care, transportation, and other service industries. These findings are in line with the results of the current study except regarding the health care industry. The difference may be explained by pandemic timing; Hawkins^3^ reported using US data from June 2020, a relatively early phase of the pandemic when the health care sector was less well prepared and experiencing a shortage of personal protective equipment. Moreover, the inverse association between COVID-19 outcomes and percentage of workers in the health care industry was not monotonic nor consistent across quintile categories in the current study, suggesting that this association may be confounded by other unobserved factors. We observed that the steepest socioeconomic disparities were associated with the proportion of workers in the restaurant industry. This finding is consistent with the report that the risk of COVID-19 transmission was high at restaurants, particularly those with indoor dining.^28,29^

Previous studies found an association between lower educational attainment and a higher burden of COVID-19.^5,6^ However, we found an inverse, but not monotonic, association; this difference may be attributed to the lack of granularity in the data we used.

Some studies showed a direct association between household crowding and COVID-19 outcomes.^4,5^ For example, Chen and Krieger^4^ reported a nearly 3-fold COVID-19 mortality rate in US counties with the highest household crowding compared with counties with the lowest crowding. In the present study, a similar association was observed after adjusting for prefecture-level covariates, but the association for mortality RR turned null after further adjusting for other socioeconomic variables, whereas the association for incidence RR remained. This finding suggests that the observed association between household crowding and COVID-19 mortality was confounded by other socioeconomic variables (household income adjusted by regional price parities, Gini coefficient, the proportion of the population receiving public assistance, educational attainment, and unemployment rate). Alternatively, the smoking rate and obesity rate, which are generally considered to be mediating variables between socioeconomic status and COVID-19 outcomes, remained associated with the COVID-19 mortality rate. This finding suggests that these variables are directly associated with COVID-19 mortality.

Several studies from Brazil showed an association between economic inequality and the COVID-19 burden,^10^ but we did not find such an association in Japan. This result may be explained by the relatively narrower economic inequality in Japan compared with Brazil (Gini coefficient: 0.36 in Japan^18^ vs 0.54 in Brazil^30^) or the relatively small variation in Gini coefficient between the prefectures in Japan (eTable 2 in the Supplement), suggesting that the associations of income inequality are observed at higher levels of spatial aggregation. Income inequality may turn out to be a factor in the COVID-19 burden from a cross-national perspective but less consistently associated with COVID-19 outcomes within countries. Another possibility is that the differences in health care systems and public health measures play a role in contrasting results. Further studies are needed to examine the potential association in other countries.

The current study showed an association between the proportion of the population receiving public assistance and COVID-19 outcomes. The mediation analysis showed that the association for COVID-19 mortality turned null after including smoking rate among prefectures with the highest percentage of public assistance recipients, suggesting that the association is mediated by the smoking rate. These findings are consistent with the report that the smoking rate was higher among public assistance recipients than the general public in Japan.^31^

The mechanisms through which socioeconomic circumstances are associated with the burden of COVID-19 are not completely understood. Low socioeconomic status has been associated with a higher prevalence of chronic diseases, such as diabetes and cardiovascular disease,^32,33^ which are risk factors for severe COVID-19. One study has suggested that low socioeconomic status is correlated with increased inflammatory responses and impaired immune response, which may play a role in the disparities in COVID-19 outcomes.^34^ Several studies have also shown that communities with social disadvantages have less ability to maintain physical distancing because of crowded housing, greater reliance on public transportation, and higher engagement in customer-facing occupations.^35,36^

The East Asian region was widely considered successful in controlling the first waves of the pandemic.^37^ On closer examination, however, we found a pattern of unequal burden associated with socioeconomic circumstances in Japan that was similar to that reported in Western countries. This finding suggests that COVID-19 outcome disparities are not a unique problem to the US and Europe. Socioeconomic circumstances also were factors in previous pandemics (eg, bubonic plague and 1918 influenza), underscoring the enduring pattern of health inequities in society.^38^

At the time of this writing, the Ministry of Health, Labour and Welfare of Japan was launching the national COVID-19 vaccination campaign. Vaccination priority groups included health care workers, older adults, and people with preexisting conditions. The results of this study suggest that vaccination and other COVID-19 planning need to prioritize populations in socially disadvantaged regions as well.

### Limitations

This study has several limitations. First, because this was an ecological study that evaluated the association between social determinants and the COVID-19 burden, we could not discount the possibility of the ecological fallacy (ie, that individual-level associations may differ in magnitude and direction from group-level associations). Moreover, the cross-sectional design of the study precluded causal inferences. Second, we conducted a prefectural ecological study because the most granular data available for COVID-19 outcomes and socioeconomic characteristics were at the prefecture level. The prefecture-level analysis averages out the differences in socioeconomic characteristics in smaller areas, such as cities or towns. Further analyses are needed to collect more granular-level data on COVID-19 and social determinants. Several US studies were able to analyze at the zip code level or census block group level by using data sources, such as the US Census Bureau and American Community Survey.^4,39^ Ultimately, individual-level data need to be collected, which will require changes to COVID-19 reporting systems and electronic health records.^38^

Third, we compared the crude rates of COVID-19 outcomes because disaggregated data were not available. Therefore, our data could still be confounded by the differences in sex and age of the populations between prefectures, although indirect standardization analysis did not suggest large differences. Fourth, the number of observed COVID-19 cases can be altered by test accessibility. Accessibility to testing can be addressed by the density of acute care hospital beds or PCR tests per population, as we did in this study, but other factors may play a role in access. If the accessibility was associated with factors, such as income level, we may have underestimated the association. Fifth, we did not control for prefecture-level differences in public health interventions and COVID-19–related behaviors (eg, public transportation use). Although the direction of bias was unclear, we cannot rule out the possibility that the omission of these variables might have biased the study results. Sixth, we extracted obesity data from the Specific Health Checkup program in Japan. In 2017, 53.1% of the targeted population received the checkup,^40^ and these people could have been more health oriented. This possible sampling bias could have biased the association between COVID-19 outcomes and the obesity rate.

## Conclusions

This cross-sectional study suggests that the burden of COVID-19 in Japan was associated with social disparities. A similar pattern of unequal burden associated with socioeconomic circumstances has been reported in Western countries. A national COVID-19 response should also prioritize the populations in socially disadvantaged regions.

## Notes

Supplement. **eTable 1.** Data Source of the Japanese Prefecture-Level Socioeconomic Characteristics and Other Covariates **eTable 2.** Socioeconomic Characteristics Data in 47 Prefectures, Japan **eTable 3.** Cut-off Values for Quintiles of the Japanese Prefecture-Level Socioeconomic Characteristics **eTable 4.** Data of COVID-19 Case, Death, Incidence Rate and Mortality Rate in 47 Prefectures, Japan, as of February 13, 2021 **eTable 5.** Japanese COVID-19 Incidence Rate Ratio and Mortality Rate Ratio by Prefectural Socioeconomic Characteristics, Further Adjusted for Household Crowding, Smoking Rate, or Obesity Rate, as of February 13, 2021 **eTable 6.** Japanese COVID-19 Incidence Rate, Mortality Rate, Incidence Rate Ratio, and Mortality Rate Ratio by Prefectural Socioeconomic Characteristics, Further Adjusted for PCR Tests per Population, as of February 13, 2021 **eTable 7.** Japanese COVID-19 Incidence Rate Ratio and Mortality Rate Ratio by Prefectural Socioeconomic Characteristics, with Sex- and Age-Adjusted by Indirect Standardization, as of February 13, 2021 eReferences
