# Supplementary material for: Association of Socioeconomic Characteristics With Disparities in COVID-19 Outcomes in Japan
Source: JAMA Netw Open. 2021 Jul 14;4(7):e2117060. doi: 10.1001/jamanetworkopen.2021.17060 (PMC8281007; doi:10.1001/jamanetworkopen.2021.17060)
Supplement: Supplement. — eTable 1. Data Source of the Japanese Prefecture-Level Socioeconomic Characteristics and Other Covariates eTable 2. Socioeconomic Characteristics Data in 47 Prefectures, Japan eTable 3. Cut-off Values for Quintiles of the Japanese Prefecture-Level Socioeconomic Characteristics eTable 4. Data of COVID-19 Case, Death, Incidence Rate and Mortality Rate in 47 Prefectures, Japan, as of February 13, 2021 eTable 5. Japanese COVID-19 Incidence Rate Ratio and Mortality Rate Ratio by Prefectural Socioeconomic Characteristics, Further Adjusted for Household Crowding, Smoking Rate, or Obesity Rate, as of February 13, 2021 eTable 6. Japanese COVID-19 Incidence Rate, Mortality Rate, Incidence Rate Ratio, and Mortality Rate Ratio by Prefectural Socioeconomic Characteristics, Further Adjusted for PCR Tests per Population, as of February 13, 2021 eTable 7. Japanese COVID-19 Incidence Rate Ratio and Mortality Rate Ratio by Prefectural Socioeconomic Characteristics, with Sex- and Age-Adjusted by Indirect Standardization, as of February 13, 2021 eReferences [file jamanetwopen-e2117060-s001.pdf]

Supplementary Online Content

Yoshikawa Y, Kawachi I. Association of socioeconomic characteristics with disparities in COVID-19 outcomes in Japan. *JAMA Netw Open*. 2021;4(7):e2117060. doi:10.1001/jamanetworkopen.2021.17060

eTable 1. Data Source of the Japanese Prefecture-Level Socioeconomic Characteristics and Other Covariates

eTable 2. Socioeconomic Characteristics Data in 47 Prefectures, Japan

eTable 3. Cut-off Values for Quintiles of the Japanese Prefecture-Level Socioeconomic Characteristics

eTable 4. Data of COVID-19 Case, Death, Incidence Rate and Mortality Rate in 47 Prefectures, Japan, as of February 13, 2021

eTable 5. Japanese COVID-19 Incidence Rate Ratio and Mortality Rate Ratio by Prefectural Socioeconomic Characteristics, Further Adjusted for Household Crowding, Smoking Rate, or Obesity Rate, as of February 13, 2021

eTable 6. Japanese COVID-19 Incidence Rate, Mortality Rate, Incidence Rate Ratio, and Mortality Rate Ratio by Prefectural Socioeconomic Characteristics, Further Adjusted for PCR Tests per Population, as of February 13, 2021

eTable 7. Japanese COVID-19 Incidence Rate Ratio and Mortality Rate Ratio by Prefectural Socioeconomic Characteristics, with Sex- and Age-Adjusted by Indirect Standardization, as of February 13, 2021

eReferences

This supplementary material has been provided by the authors to give readers additional information about their work.

© 2021 Yoshikawa Y et al. *JAMA Network Open.*

eTable 1. Data Source of the Japanese Prefecture-Level Socioeconomic Characteristics and Other Covariates

Category	Variables	Data source	Year
Income and wealth	Mean annual household income adjusted by regional price parities (thousand JPY)	National Survey of Family Income and Expenditure[1] and Retail Price Survey[2]	2014
	Gini coefficient	National Survey of Family Income and Expenditure[1]	2014
	Proportion of the population receiving public assistance (average monthly number of public assistance recipients divided by total population) (per 1,000 population)	National Survey on Public Assistance Recipients[3]	2018
Educational attainment	Percentage of graduates aged 20 years or older with a college or a higher-level degree	Employment Status Survey[4]	2017
Occupation and unemployment	Unemployment rate	Labour Force Survey[5]	2019
	Percentage of workers employed in<br>- Health care (medical, health care, and welfare) industry<br>- Retail (retail trade) industry<br>- Transportation and postal (transport and postal service) industry<br>- Restaurant (eating and drinking places) industry	Employment Status Survey[4]	2017
Living environment	Household crowding (tatami units of living area (total living space including living rooms, bedrooms, studies and dining rooms, etc., and excluding entrance halls, kitchens, toilet rooms, bathrooms, corridor, etc.) per person) (tatami units per person)[a]	Housing and Land Survey[6]	2018
Health-related factors	Smoking rate (Percentage of those who smoke among people aged 20 years and over)	Comprehensive Survey of Living Conditions[7]	2019
	Obesity rate (Percentage of those whose BMI is 25 or above among people aged 40-74 years)	NDB Open Data Japan[8]	2017
Other variables	Percentage of the older adult population (Percentage of the population aged 65 and over)	Population Estimates[9]	2019
	Population density (population per square kilometer)	Population Census[10]	2015
	Number of acute care hospital beds per population (number of highly-acute and acute hospital beds divided per population) (per 1,000 population)	Hospital Bed Function Report[11] and Population Estimates[9]	2018

[a] 1.65 square meters per person

© 2021 Yoshikawa Y et al. *JAMA Network Open.*

eTable 2. Socioeconomic Characteristics Data in 47 Prefectures, Japan

	Prefecture	Household income adjusted by regional price parities	Gini coefficient	Proportion of the population receiving public assistance	Educational attainment: college or higher-level degree	Unemployment rate	Percentage of workers in health care industry	Percentage of workers in retail industry	Percentage of workers in transportation and postal industry
1	Hokkaido	4,643	0.36	23.4	16.2	2.6	13.9	12.0	5.9
2	Aomori	4,505	0.374	22.0	12.3	2.7	13.2	11.7	4.5
3	Iwate	5,100	0.347	9.0	12.9	2.1	12.9	11.0	4.9
4	Miyagi	5,086	0.383	8.9	19.8	2.5	11.8	11.6	5.7
5	Akita	5,264	0.334	13.5	11.4	2.8	14.8	11.7	3.7
6	Yamagata	5,781	0.376	7.2	13.6	1.7	12.8	11.3	3.4
7	Fukushima	5,085	0.358	7.4	13.8	2.1	11.8	10.5	4.6
8	Ibaraki	5,774	0.343	9.7	18.6	2.3	10.1	10.5	5.4
9	Tochigi	5,632	0.351	8.5	17.5	2.2	10.8	10.8	4.5
10	Gumma	5,612	0.35	6.1	17.6	2.3	12.2	10.5	4.7
11	Saitama	5,616	0.349	12.0	26.3	2.3	10.7	10.9	7.0
12	Chiba	5,775	0.334	12.2	28.1	2.1	10.9	10.9	6.8
13	Tokyo	5,702	0.378	21.0	41.5	2.3	10.1	9.3	4.7
14	Kanagawa	5,562	0.35	12.4	32.9	2.1	11.0	11.1	5.9
15	Niigata	5,481	0.335	6.2	14.7	2.1	12.5	11.5	4.6
16	Toyama	6,127	0.338	2.6	20.4	1.7	11.9	11.0	4.3
17	Ishikawa	5,692	0.348	4.4	20.8	1.6	12.2	11.8	4.3
18	Fukui	6,290	0.34	5.3	18.5	1.4	12.4	11.0	3.6
19	Yamanashi	5,696	0.341	8.6	20.7	2	11.4	11.5	3.8
20	Nagano	5,527	0.317	4.6	18.0	1.9	12.2	10.5	3.6
21	Gifu	5,811	0.344	3.3	19.5	1.3	11.8	11.1	4.1
22	Shizuoka	5,867	0.341	6.8	19.7	2	10.8	10.6	5.1
23	Aichi	5,753	0.35	5.7	25.5	1.9	10.4	10.6	5.4
24	Mie	5,705	0.332	8.9	19.3	1.2	12.2	10.7	4.9
25	Shiga	5,850	0.333	6.7	23.8	1.9	12.1	11.0	4.5
26	Kyoto	4,901	0.362	12.8	28.2	2.3	13.6	12.3	4.3
27	Osaka	4,885	0.372	20.4	25.8	2.9	12.8	10.1	6.1
28	Hyogo	5,148	0.346	8.3	28.0	2.3	13.3	11.1	5.3
29	Nara	5,613	0.333	12.9	28.9	1.9	14.5	11.6	3.5
30	Wakayama	4,786	0.374	10.3	17.4	1.6	15.2	12.1	4.3
31	Tottori	5,335	0.364	11.0	17.0	2.3	15.8	10.6	3.6
32	Shimane	5,210	0.361	6.3	15.8	1.9	16.4	11.9	3.3
33	Okayama	5,216	0.342	6.8	21.8	2.3	14.2	10.3	5.2
34	Hiroshima	5,047	0.348	8.2	24.8	2.4	13.3	11.3	5.2
35	Yamaguchi	4,780	0.361	9.7	17.5	1.8	15.2	12.1	4.7
36	Tokushima	5,052	0.369	18.3	20.6	1.9	16.5	10.6	3.6
37	Kagawa	5,040	0.349	8.0	21.7	2	13.6	11.0	4.4
38	Ehime	4,716	0.353	11.1	19.7	1.6	14.9	11.3	4.6
39	Kochi	4,687	0.363	19.3	15.7	1.9	16.9	11.5	3.5
40	Fukuoka	4,832	0.362	22.9	22.7	2.8	14.6	11.1	5.8
41	Saga	5,209	0.344	9.6	16.1	1.9	14.6	10.4	4.5
42	Nagasaki	4,603	0.362	15.5	14.3	2.2	17.5	11.1	4.5
43	Kumamoto	4,975	0.36	9.2	17.6	2.7	16.8	10.5	3.7
44	Oita	4,721	0.377	16.8	16.1	2	15.7	11.9	3.9
45	Miyazaki	4,710	0.342	13.4	14.3	1.6	16.4	11.3	4.1
46	Kagoshima	4,490	0.378	15.1	14.0	2.4	17.1	11.6	4.0
47	Okinawa	3,914	0.366	21.7	18.8	2.7	15.5	10.5	4.3

© 2021 Yoshikawa Y et al. *JAMA Network Open.*

eTable 2. Socioeconomic Characteristics Data in 47 Prefectures, Japan (continued)

	Prefecture	Percentage of workers in restaurant industry	Household crowding	Smoking rate	Obesity rate	Percentage of the older adult population	Population density	Number of acute care hospital beds per population
1	Hokkaido	3.7	15.6	22.6	31.2	31.9	68.6	7.45
2	Aomori	2.7	16.6	22.2	31.6	33.3	135.6	6.10
3	Iwate	3.3	16.1	20.9	31.6	33.1	83.8	5.63
4	Miyagi	3.6	14.0	21.0	30.8	28.3	320.5	5.39
5	Akita	3.1	17.7	20.7	30.8	37.2	87.9	6.49
6	Yamagata	3.3	15.8	18.1	29.1	33.4	120.5	5.82
7	Fukushima	3.4	15.1	21.9	31.6	31.5	138.9	6.40
8	Ibaraki	3.7	14.6	19.2	29.9	29.5	478.4	4.78
9	Tochigi	3.5	14.3	19.7	29.6	28.6	308.1	5.13
10	Gumma	3.7	14.7	19.5	27.9	29.9	310.1	5.62
11	Saitama	4.2	13.2	18.3	28.3	26.7	1913.4	4.01
12	Chiba	3.9	13.8	20.2	28.7	27.8	1206.5	4.66
13	Tokyo	4.5	12.2	16.5	26.5	23.1	6168.7	4.85
14	Kanagawa	4.5	13.0	17.3	27.1	25.3	3777.7	4.15
15	Niigata	3.8	16.0	18.7	25.2	32.4	183.1	5.52
16	Toyama	3.0	17.4	17.9	26.7	32.3	251	5.87
17	Ishikawa	4.1	16.7	17.2	26.2	29.6	275.7	6.60
18	Fukui	3.2	15.7	18.6	26.5	30.6	187.7	6.21
19	Yamanashi	3.7	15.4	18.8	25.4	30.8	187	5.74
20	Nagano	3.2	16.0	16.9	25.3	31.9	154.8	5.73
21	Gifu	3.9	15.9	17.0	25.2	30.1	191.3	5.05
22	Shizuoka	3.4	14.2	18.4	25.8	29.9	475.8	4.61
23	Aichi	4.3	14.0	18.1	26.8	25.1	1446.7	4.38
24	Mie	3.6	15.6	18.2	27.1	29.7	314.5	5.07
25	Shiga	3.7	15.4	16.1	25.6	26.0	351.7	4.58
26	Kyoto	5.1	13.8	15.8	24.6	29.2	566	6.05
27	Osaka	4.6	12.8	19.1	27.0	27.6	4639.8	5.83
28	Hyogo	3.7	14.5	15.6	25.7	29.1	658.8	5.47
29	Nara	3.7	15.5	15.1	26.3	31.3	369.6	6.05
30	Wakayama	3.5	14.8	17.4	27.0	33.1	203.9	6.72
31	Tottori	3.0	15.1	17.6	25.2	32.0	163.5	6.44
32	Shimane	2.8	15.1	15.9	24.6	34.3	103.5	6.16
33	Okayama	3.2	14.7	17.4	27.4	30.3	270.1	6.52
34	Hiroshima	3.7	14.6	17.5	27.6	29.4	335.4	5.81
35	Yamaguchi	3.4	15.3	16.8	25.7	34.3	229.8	6.29
36	Tokushima	3.0	15.7	16.8	30.2	33.7	182.3	5.77
37	Kagawa	3.6	16.1	16.5	29.0	31.9	520.2	6.66
38	Ehime	3.1	14.9	15.8	29.4	33.0	244.1	6.69
39	Kochi	3.5	15.0	18.2	29.3	35.2	102.5	7.84
40	Fukuoka	4.4	13.5	19.8	27.0	27.9	1023.1	6.20
41	Saga	3.0	14.2	21.0	27.4	30.2	341.2	5.90
42	Nagasaki	3.6	13.7	19.0	28.7	32.7	333.3	6.66
43	Kumamoto	3.5	13.5	18.5	28.9	31.1	241.1	5.79
44	Oita	3.5	14.7	17.7	29.4	32.9	183.9	7.82
45	Miyazaki	3.4	13.9	18.8	29.5	32.2	142.7	6.76
46	Kagoshima	3.6	13.5	19.0	30.6	31.9	179.4	7.00
47	Okinawa	4.6	11.1	19.8	38.9	22.2	628.4	5.02

© 2021 Yoshikawa Y et al. *JAMA Network Open.*

eTable 3. Cut-off Values for Quintiles of the Japanese Prefecture-Level Socioeconomic Characteristics

	Quintile				
	1st	2nd	3rd	4th	5th
Household income adjusted by regional price parities (thousand JPY)	3,913 - 4,779	4,780 - 5,084	5,085 - 5,479	5,480 - 5,704	5,705 - 6,290
Gini coefficient	0.317 - 0.341	0.342 - 0.348	0.349 - 0.359	0.360 - 0.365	0.366 - 0.383
Proportion of the population receiving public assistance (per 1,000 population)	2.6 - 6.7	6.8 - 8.8	8.9 - 11.0	11.1 - 15.4	15.5 - 23.4
Educational attainment: college or higher-level degree (%)	11.4 - 15.6	15.7 - 17.5	17.6 - 19.7	19.7 - 24.7	24.8 - 41.5
Unemployment rate (%)	1.2 - 1.8	1.9 - 2.0	2.1 - 2.2	2.3 - 2.3	2.4- 2.9
Percentage of workers in health care industry (%)	10.1 - 11.8	11.8 - 12.7	12.8 - 14.1	14.2 - 15.6	15.7 - 17.5
Percentage of workers in retail industry (%)	9.3 - 10.5	10.6 - 11.0	11.0 - 11.3	11.3 - 11.6	11.7 - 12.3
Percentage of workers in transportation and postal industry (%)	3.3 - 3.7	3.8 - 4.3	4.4 - 4.6	4.7 - 5.3	5.4 - 7.0
Percentage of workers in restaurant industry (%)	2.7 - 3.2	3.2 - 3.5	3.5 - 3.7	3.7 - 4.0	4.1 - 5.1
Household crowding (tatami units per person)[a]	11.1 - 13.7	13.8 - 14.5	14.6 - 15.1	15.1 - 15.7	15.8 - 17.7
Smoking rate (%)	15.1 - 16.8	16.9 - 18.0	18.1 - 18.7	18.8 - 19.8	19.8 - 22.6
Obesity rate (%)	24.6 - 25.7	25.8 - 27.0	27.1 - 28.8	28.9 - 30.1	30.2 - 38.9

[a] 1.65 square meters per person

© 2021 Yoshikawa Y et al. *JAMA Network Open.*

eTable 4. Data of COVID-19 Case, Death, Incidence Rate and Mortality Rate in 47 Prefectures, Japan, as of February 13, 2021[12]

		Case	Death	Population (1,000 people)	Incidence Rate per 100 000	Mortality Rate per 100 000
Overall		412,275	6,910	126,167	326.8	5.5
Prefectures						
1	Hokkaido	18,441	637	5,250	351.3	12.1
2	Aomori	801	16	1,246	64.3	1.3
3	Iwate	520	29	1,227	42.4	2.4
4	Miyagi	3,493	23	2,306	151.5	1.0
5	Akita	269	5	966	27.8	0.5
6	Yamagata	532	15	1,078	49.4	1.4
7	Fukushima	1,827	63	1,846	99.0	3.4
8	Ibaraki	5,334	91	2,860	186.5	3.2
9	Tochigi	3,954	58	1,934	204.4	3.0
10	Gumma	4,231	79	1,942	217.9	4.1
11	Saitama	27,633	459	7,350	376.0	6.2
12	Chiba	24,476	344	6,259	391.1	5.5
13	Tokyo	106,134	1125	13,921	762.4	8.1
14	Kanagawa	43,156	596	9,198	469.2	6.5
15	Niigata	1,003	13	2,223	45.1	0.6
16	Toyama	887	27	1,044	85.0	2.6
17	Ishikawa	1,652	60	1,138	145.2	5.3
18	Fukui	530	24	768	69.0	3.1
19	Yamanashi	919	16	811	113.3	2.0
20	Nagano	2,345	39	2,049	114.4	1.9
21	Gifu	4,554	89	1,987	229.2	4.5
22	Shizuoka	4,824	92	3,644	132.4	2.5
23	Aichi	25,069	471	7,552	332.0	6.2
24	Mie	2,379	42	1,781	133.6	2.4
25	Shiga	2,305	39	1,414	163.0	2.8
26	Kyoto	8,846	144	2,583	342.5	5.6
27	Osaka	45,849	1052	8,809	520.5	11.9
28	Hyogo	17,415	476	5,466	318.6	8.7
29	Nara	3,254	43	1,330	244.7	3.2
30	Wakayama	1,143	16	925	123.6	1.7
31	Tottori	207	2	556	37.2	0.4
32	Shimane	280	0	674	41.5	0
33	Okayama	2,442	26	1,890	129.2	1.4
34	Hiroshima	4,931	98	2,804	175.9	3.5
35	Yamaguchi	1,352	33	1,358	99.6	2.4
36	Tokushima	424	15	728	58.2	2.1
37	Kagawa	717	17	956	75.0	1.8
38	Ehime	1,029	22	1,339	76.8	1.6
39	Kochi	874	17	698	125.2	2.4
40	Fukuoka	17,263	230	5,104	338.2	4.5
41	Saga	995	6	815	122.1	0.7
42	Nagasaki	1,580	35	1,327	119.1	2.6
43	Kumamoto	3,403	67	1,748	194.7	3.8
44	Oita	1,261	18	1,135	111.1	1.6
45	Miyazaki	1,906	20	1,073	177.6	1.9
46	Kagoshima	1,716	21	1,602	107.1	1.3
47	Okinawa	7,971	100	1,453	548.6	6.9

© 2021 Yoshikawa Y et al. *JAMA Network Open.*

eTable 5. Japanese COVID-19 Incidence Rate Ratio and Mortality Rate Ratio by Prefectural Socioeconomic Characteristics, Further Adjusted for Household Crowding, Smoking Rate, and/or Obesity Rate, as of February 13, 2021

Socioeconomic variable	Incidence RR (95% CI)[a]				Mortality RR (95% CI)[a]			
	Model 2[b]	+ Household Crowding	+ Smoking Rate	+ Obesity Rate	Model 2[b]	+ Household Crowding	+ Smoking Rate	+ Obesity Rate
Household income adjusted by regional price parities								
5th quintile	1 [Reference]	1 [Reference]	1 [Reference]	1 [Reference]	1 [Reference]	1 [Reference]	1 [Reference]	1 [Reference]
4th quintile	0.99 (0.98-1.00)	0.97 (0.96-0.98)	1.04 (1.02-1.05)	1.00 (0.99-1.01)	0.78 (0.71-0.85)	0.85 (0.78-0.93)	0.84 (0.77-0.91)	0.79 (0.72-0.86)
3rd quintile	1.02 (1.00-1.03)	0.96 (0.94-0.97)	1.03 (1.01-1.04)	1.07 (1.05-1.09)	1.17 (1.05-1.30)	1.47 (1.31-1.66)	1.19 (1.06-1.32)	1.27 (1.13-1.42)
2nd quintile	1.31 (1.30-1.33)	1.19 (1.17-1.21)	1.29 (1.27-1.30)	1.38 (1.36-1.39)	1.34 (1.22-1.49)	1.97 (1.74-2.24)	1.33 (1.20-1.47)	1.44 (1.30-1.61)
1st quintile	1.45 (1.43-1.48)	1.28 (1.25-1.31)	1.33 (1.31-1.36)	1.73 (1.68-1.77)	1.81 (1.59-2.07)	2.91 (2.48-3.42)	1.62 (1.41-1.85)	2.39 (2.00-2.85)
Gini coefficient								
1st quintile	1 [Reference]	1 [Reference]	1 [Reference]	1 [Reference]	1 [Reference]	1 [Reference]	1 [Reference]	1 [Reference]
2nd quintile	1.05 (1.04-1.07)	1.00 (0.98-1.01)	1.20 (1.18-1.22)	1.06 (1.05-1.08)	1.32 (1.19-1.47)	1.32 (1.18-1.47)	1.61 (1.44-1.79)	1.34 (1.21-1.49)
3rd quintile	0.96 (0.95-0.97)	0.89 (0.87-0.90)	0.93 (0.92-0.94)	0.89 (0.88-0.90)	1.25 (1.13-1.37)	1.24 (1.13-1.37)	1.20 (1.09-1.32)	1.13 (1.03-1.25)
4th quintile	1.34 (1.32-1.37)	1.07 (1.05-1.09)	1.34 (1.31-1.36)	1.33 (1.31-1.36)	1.38 (1.22-1.56)	1.36 (1.19-1.56)	1.33 (1.17-1.51)	1.36 (1.20-1.54)
5th quintile	0.81 (0.80-0.83)	0.63 (0.62-0.65)	0.77 (0.75-0.78)	0.68 (0.66-0.69)	0.79 (0.69-0.90)	0.78 (0.67-0.91)	0.74 (0.64-0.85)	0.66 (0.57-0.77)
Proportion of the population receiving public assistance								
1st quintile	1 [Reference]	1 [Reference]	1 [Reference]	1 [Reference]	1 [Reference]	1 [Reference]	1 [Reference]	1 [Reference]
2nd quintile	1.11 (1.10-1.13)	1.13 (1.11-1.14)	1.13 (1.12-1.15)	1.16 (1.15-1.18)	1.11 (1.01-1.22)	1.20 (1.08-1.34)	1.14 (1.03-1.25)	1.11 (1.01-1.23)
3rd quintile	0.92 (0.91-0.94)	0.93 (0.92-0.95)	0.89 (0.87-0.91)	1.02 (1.00-1.04)	0.63 (0.55-0.72)	0.70 (0.61-0.81)	0.57 (0.50-0.65)	0.64 (0.55-0.73)
4th quintile	1.55 (1.54-1.57)	1.58 (1.56-1.60)	1.51 (1.49-1.52)	1.70 (1.67-1.72)	1.23 (1.13-1.34)	1.39 (1.25-1.54)	1.12 (1.03-1.22)	1.23 (1.12-1.36)
5th quintile	1.55 (1.52-1.58)	1.58 (1.55-1.61)	1.37 (1.34-1.39)	1.80 (1.77-1.84)	1.51 (1.35-1.69)	1.70 (1.49-1.94)	1.09 (0.96-1.24)	1.52 (1.33-1.74)
Educational attainment: college or higher-level degree								
5th quintile	1 [Reference]	1 [Reference]	1 [Reference]	1 [Reference]	1 [Reference]	1 [Reference]	1 [Reference]	1 [Reference]
4th quintile	0.58 (0.57-0.58)	0.60 (0.59-0.60)	0.55 (0.54-0.56)	0.58 (0.57-0.58)	0.38 (0.34-0.42)	0.38 (0.34-0.42)	0.34 (0.31-0.38)	0.37 (0.34-0.41)
3rd quintile	0.70 (0.69-0.71)	0.68 (0.67-0.69)	0.68 (0.67-0.69)	0.61 (0.60-0.62)	0.54 (0.49-0.60)	0.54 (0.49-0.60)	0.51 (0.46-0.56)	0.45 (0.40-0.50)
2nd quintile	0.78 (0.77-0.80)	0.82 (0.80-0.83)	0.66 (0.65-0.67)	0.69 (0.67-0.70)	0.66 (0.59-0.74)	0.66 (0.59-0.74)	0.46 (0.41-0.53)	0.53 (0.47-0.60)
1st quintile	0.37 (0.37-0.38)	0.37 (0.36-0.38)	0.33 (0.32-0.33)	0.31 (0.30-0.32)	0.26 (0.22-0.30)	0.26 (0.22-0.30)	0.20 (0.17-0.23)	0.19 (0.16-0.23)
Unemployment rate								
1st quintile	1 [Reference]	1 [Reference]	1 [Reference]	1 [Reference]	1 [Reference]	1 [Reference]	1 [Reference]	1 [Reference]
2nd quintile	1.03 (1.01-1.05)	1.03 (1.01-1.05)	1.01 (0.99-1.03)	0.99 (0.97-1.01)	1.08 (0.95-1.23)	1.10 (0.96-1.25)	1.04 (0.92-1.19)	1.06 (0.93-1.21)
3rd quintile	1.41 (1.38-1.44)	1.39 (1.36-1.41)	1.34 (1.32-1.37)	1.45 (1.42-1.47)	1.31 (1.15-1.49)	1.46 (1.28-1.66)	1.16 (1.02-1.33)	1.35 (1.19-1.54)
4th quintile	1.40 (1.38-1.43)	1.38 (1.36-1.41)	1.38 (1.36-1.41)	1.41 (1.38-1.43)	1.38 (1.22-1.57)	1.53 (1.35-1.74)	1.33 (1.18-1.51)	1.39 (1.23-1.58)
5th quintile	1.56 (1.53-1.59)	1.52 (1.49-1.55)	1.44 (1.42-1.47)	1.64 (1.61-1.67)	1.85 (1.65-2.09)	2.19 (1.93-2.50)	1.56 (1.37-1.79)	1.93 (1.71-2.18)
Percentage of workers in health care industry								
1st quintile	1 [Reference]	1 [Reference]	1 [Reference]	1 [Reference]	1 [Reference]	1 [Reference]	1 [Reference]	1 [Reference]
2nd quintile	0.68 (0.67-0.69)	0.73 (0.72-0.74)	0.72 (0.71-0.73)	0.69 (0.68-0.70)	0.65 (0.58-0.72)	0.67 (0.60-0.75)	0.72 (0.64-0.81)	0.67 (0.60-0.75)
3rd quintile	1.27 (1.25-1.28)	1.10 (1.09-1.12)	1.31 (1.30-1.33)	1.27 (1.25-1.28)	1.72 (1.58-1.87)	1.63 (1.48-1.78)	1.92 (1.76-2.10)	1.75 (1.61-1.91)
4th quintile	0.91 (0.89-0.92)	0.71 (0.69-0.72)	0.94 (0.93-0.96)	0.90 (0.89-0.92)	0.61 (0.54-0.68)	0.55 (0.48-0.63)	0.67 (0.59-0.75)	0.60 (0.54-0.68)
5th quintile	0.77 (0.75-0.79)	0.53 (0.51-0.54)	0.86 (0.84-0.88)	0.77 (0.75-0.79)	0.55 (0.46-0.65)	0.46 (0.38-0.57)	0.70 (0.58-0.84)	0.55 (0.46-0.66)

Abbreviations: RR, rate ratio.

[a] Incidence and mortality rate ratios were calculated using Poisson regression models with log(population) as the offset.

[b] Model 2 is controlled for each socioeconomic characteristic and prefecture-level characteristics (percentage of the older adult population, population density, and number of acute care hospital beds per population).

© 2021 Yoshikawa Y et al. *JAMA Network Open.*

eTable 5. Japanese COVID-19 Incidence Rate Ratio and Mortality Rate Ratio by Prefectural Socioeconomic Characteristics, Further Adjusted for Household Crowding, Smoking Rate, and/or Obesity Rate, as of February 13, 2021 (continued)

	Incidence RR (95% CI)[a]					Mortality RR (95% CI)[a]			
Socioeconomic variable	Model 2[b]	+ Household Crowding	+ Smoking Rate	+ Obesity Rate		Model 2[b]	+ Household Crowding	+ Smoking Rate	+ Obesity Rate
Percentage of workers in retail industry									
1st quintile	1 [Reference]	1 [Reference]	1 [Reference]	1 [Reference]		1 [Reference]	1 [Reference]	1 [Reference]	1 [Reference]
2nd quintile	1.25 (1.23-1.27)	1.33 (1.31-1.35)	1.19 (1.18-1.21)	1.32 (1.30-1.34)		1.44 (1.30-1.59)	1.46 (1.32-1.62)	1.34 (1.21-1.49)	1.50 (1.36-1.67)
3rd quintile	1.19 (1.17-1.20)	1.24 (1.22-1.25)	1.22 (1.20-1.23)	1.25 (1.24-1.27)		1.30 (1.20-1.41)	1.31 (1.21-1.43)	1.35 (1.24-1.46)	1.39 (1.28-1.51)
4th quintile	0.77 (0.76-0.79)	0.79 (0.77-0.80)	0.80 (0.79-0.82)	0.79 (0.77-0.80)		0.44 (0.37-0.51)	0.44 (0.37-0.51)	0.47 (0.40-0.55)	0.44 (0.38-0.52)
5th quintile	1.36 (1.34-1.38)	1.54 (1.51-1.56)	1.40 (1.38-1.42)	1.40 (1.38-1.42)		1.45 (1.31-1.61)	1.52 (1.37-1.69)	1.47 (1.33-1.63)	1.48 (1.34-1.64)
Percentage of workers in transportation and postal industry									
1st quintile	1 [Reference]	1 [Reference]	1 [Reference]	1 [Reference]		1 [Reference]	1 [Reference]	1 [Reference]	1 [Reference]
2nd quintile	1.27 (1.24-1.30)	1.22 (1.20-1.25)	1.27 (1.25-1.30)	1.27 (1.24-1.30)		1.38 (1.17-1.63)	1.43 (1.21-1.68)	1.38 (1.17-1.63)	1.38 (1.17-1.62)
3rd quintile	0.78 (0.76-0.80)	0.79 (0.77-0.81)	0.78 (0.76-0.80)	0.77 (0.76-0.79)		0.90 (0.76-1.08)	0.90 (0.75-1.07)	0.95 (0.79-1.13)	0.90 (0.75-1.07)
4th quintile	1.29 (1.26-1.32)	1.31 (1.28-1.34)	1.29 (1.26-1.32)	1.30 (1.27-1.32)		1.78 (1.54-2.08)	1.78 (1.53-2.07)	1.83 (1.57-2.14)	1.79 (1.54-2.09)
5th quintile	1.61 (1.57-1.64)	1.58 (1.55-1.61)	1.61 (1.58-1.64)	1.61 (1.57-1.64)		2.55 (2.21-2.94)	2.60 (2.26-3.01)	2.77 (2.37-3.25)	2.54 (2.21-2.94)
Percentage of workers in restaurant industry									
1st quintile	1 [Reference]	1 [Reference]	1 [Reference]	1 [Reference]		1 [Reference]	1 [Reference]	1 [Reference]	1 [Reference]
2nd quintile	1.44 (1.40-1.48)	1.25 (1.22-1.28)	1.41 (1.38-1.45)	1.44 (1.40-1.48)		1.56 (1.30-1.89)	1.52 (1.26-1.85)	1.53 (1.27-1.85)	1.57 (1.30-1.90)
3rd quintile	1.55 (1.51-1.60)	1.41 (1.37-1.45)	1.49 (1.45-1.53)	1.57 (1.53-1.62)		1.56 (1.29-1.90)	1.53 (1.26-1.87)	1.48 (1.22-1.81)	1.60 (1.32-1.95)
4th quintile	2.82 (2.75-2.88)	2.64 (2.58-2.71)	2.69 (2.63-2.76)	2.87 (2.81-2.94)		4.19 (3.55-4.98)	4.14 (3.51-4.93)	3.89 (3.29-4.62)	4.38 (3.71-5.21)
5th quintile	2.61 (2.54-2.68)	2.26 (2.20-2.32)	2.45 (2.39-2.52)	2.70 (2.63-2.77)		4.17 (3.48-5.03)	4.07 (3.38-4.93)	3.84 (3.19-4.64)	4.55 (3.78-5.51)

Abbreviations: RR, rate ratio.

[a] Incidence and mortality rate ratios were calculated using Poisson regression models with log(population) as the offset.

[b] Model 2 is controlled for each socioeconomic characteristic and prefecture-level characteristics (percentage of the older adult population, population density, and number of acute care hospital beds per population).

© 2021 Yoshikawa Y et al. *JAMA Network Open.*

eTable 6. Japanese COVID-19 Incidence Rate, Mortality Rate, Incidence Rate Ratio, and Mortality Rate Ratio by Prefectural Socioeconomic Characteristics, Further Adjusted for PCR Tests per Population, as of February 13, 2021

		Incidence RR (95% CI)[a]				Mortality RR (95% CI)[a]		
Socioeconomic variable	Incidence rate per 100 000 (95% CI)	Model 1[b]	Model 2[c]	Model 3[d]	Mortality rate per 100 000 (95% CI)	Model 1[b]	Model 2[c]	Model 3[d]
Household income adjusted by regional price parities								
5th quintile	249.7 (247.9-251.6)	1 [Reference]	1 [Reference]	NA	4.35 (4.11-4.60)	1 [Reference]	1 [Reference]	NA
4th quintile	463.7 (461.7-465.8)	1.86 (1.84-1.87)	0.90 (0.89-0.91)	NA	5.94 (5.71-6.18)	1.37 (1.28-1.46)	0.70 (0.64-0.77)	NA
3rd quintile	174.3 (172.3-176.4)	0.70 (0.69-0.71)	1.10 (1.08-1.12)	NA	4.00 (3.69-4.33)	0.92 (0.84-1.01)	1.31 (1.17-1.46)	NA
2nd quintile	335.5 (333.2-337.8)	1.34 (1.33-1.36)	1.20 (1.18-1.22)	NA	6.68 (6.37-7.01)	1.54 (1.43-1.66)	1.23 (1.11-1.36)	NA
1st quintile	235.3 (232.8-237.7)	0.94 (0.93-0.95)	1.38 (1.35-1.40)	NA	5.86 (5.48-6.26)	1.35 (1.24-1.47)	1.78 (1.55-2.04)	NA
Gini coefficient								
1st quintile	193.8 (192.0-195.6)	1 [Reference]	1 [Reference]	NA	3.07 (2.84-3.31)	1 [Reference]	1 [Reference]	NA
2nd quintile	206.4 (204.4-208.4)	1.07 (1.05-1.08)	1.18 (1.17-1.20)	NA	4.65 (4.35-4.96)	1.51 (1.37-1.67)	1.56 (1.40-1.73)	NA
3rd quintile	335.1 (333.1-337.1)	1.73 (1.71-1.75)	1.04 (1.03-1.06)	NA	5.50 (5.24-5.76)	1.79 (1.64-1.96)	1.38 (1.25-1.52)	NA
4th quintile	270.7 (268.4-273.1)	1.40 (1.38-1.42)	1.22 (1.20-1.25)	NA	6.04 (5.70-6.39)	1.97 (1.79-2.16)	1.23 (1.08-1.39)	NA
5th quintile	510.0 (507.5-512.4)	2.63 (2.60-2.66)	0.81 (0.80-0.83)	NA	7.23 (6.94-7.53)	2.36 (2.17-2.57)	0.80 (0.69-0.92)	NA
Proportion of the population receiving public assistance								
1st quintile	206.1 (204.2-208.1)	1 [Reference]	1 [Reference]	NA	4.05 (3.78-4.33)	1 [Reference]	1 [Reference]	NA
2nd quintile	183.9 (182.0-185.7)	0.89 (0.88-0.90)	1.06 (1.04-1.07)	NA	4.21 (3.94-4.51)	1.04 (0.95-1.15)	1.06 (0.96-1.17)	NA
3rd quintile	138.7 (136.7-140.7)	0.67 (0.66-0.68)	0.97 (0.95-0.99)	NA	2.28 (2.03-2.54)	0.56 (0.49-0.64)	0.66 (0.58-0.76)	NA
4th quintile	354.2 (352.1-356.3)	1.72 (1.70-1.74)	1.44 (1.42-1.46)	NA	5.22 (4.97-5.48)	1.29 (1.19-1.40)	1.14 (1.04-1.25)	NA
5th quintile	505.7 (503.4-507.9)	2.45 (2.43-2.48)	1.26 (1.23-1.28)	NA	8.18 (7.90-8.47)	2.02 (1.88-2.18)	1.25 (1.08-1.43)	NA
Educational attainment: college or higher-level degree								
5th quintile	470.0 (468.3-471.6)	1 [Reference]	1 [Reference]	NA	7.37 (7.16-7.58)	1 [Reference]	1 [Reference]	NA
4th quintile	183.5 (181.6-185.4)	0.39 (0.39-0.39)	0.57 (0.57-0.58)	NA	2.86 (2.63-3.11)	0.39 (0.36-0.42)	0.38 (0.34-0.42)	NA
3rd quintile	199.5 (197.3-201.7)	0.42 (0.42-0.43)	0.70 (0.69-0.70)	NA	3.47 (3.19-3.77)	0.47 (0.43-0.51)	0.55 (0.50-0.60)	NA
2nd quintile	213.6 (211.1-216.1)	0.45 (0.45-0.46)	0.72 (0.71-0.73)	NA	5.90 (5.49-6.32)	0.80 (0.74-0.86)	0.62 (0.55-0.69)	NA
1st quintile	80.7 (79.1-82.2)	0.17 (0.17-0.18)	0.40 (0.39-0.40)	NA	1.72 (1.50-1.97)	0.23 (0.20-0.27)	0.27 (0.24-0.32)	NA
Unemployment rate								
1st quintile	127.8 (125.8-129.8)	1 [Reference]	1 [Reference]	NA	2.79 (2.50-3.09)	1 [Reference]	1 [Reference]	NA
2nd quintile	198.4 (196.6-200.3)	1.55 (1.52-1.58)	1.07 (1.05-1.09)	NA	3.54 (3.30-3.80)	1.27 (1.12-1.45)	1.10 (0.96-1.25)	NA
3rd quintile	318.6 (316.4-320.9)	2.49 (2.45-2.54)	1.39 (1.36-1.41)	NA	4.74 (4.47-5.02)	1.70 (1.51-1.92)	1.27 (1.12-1.44)	NA
4th quintile	471.0 (468.8-473.2)	3.69 (3.63-3.75)	1.33 (1.30-1.35)	NA	6.57 (6.31-6.84)	2.36 (2.11-2.64)	1.32 (1.16-1.50)	NA
5th quintile	332.8 (330.8-334.9)	2.60 (2.56-2.65)	1.47 (1.45-1.50)	NA	7.19 (6.89-7.49)	2.58 (2.31-2.89)	1.76 (1.56-1.99)	NA
Percentage of workers in health care industry								
1st quintile	439.4 (437.7-441.2)	1 [Reference]	1 [Reference]	NA	5.99 (5.78-6.19)	1 [Reference]	1 [Reference]	NA
2nd quintile	140.4 (138.6-142.2)	0.32 (0.32-0.32)	0.73 (0.72-0.75)	NA	2.61 (2.37-2.87)	0.44 (0.39-0.48)	0.72 (0.65-0.81)	NA
3rd quintile	333.3 (331.2-335.4)	0.76 (0.75-0.76)	1.31 (1.29-1.33)	NA	8.44 (8.11-8.78)	1.41 (1.34-1.49)	1.84 (1.69-2.01)	NA
4th quintile	235.3 (232.9-237.7)	0.54 (0.53-0.54)	0.86 (0.85-0.88)	NA	3.17 (2.89-3.46)	0.53 (0.48-0.58)	0.60 (0.53-0.67)	NA
5th quintile	122.1 (119.9-124.4)	0.28 (0.27-0.28)	0.84 (0.82-0.86)	NA	2.04 (1.77-2.35)	0.34 (0.29-0.39)	0.63 (0.53-0.76)	NA

Abbreviations: NA, not applicable; RR, rate ratio.

[a] Incidence and mortality rate ratios were calculated using Poisson regression models with log(population) as the offset.

[b] Model 1 is controlled for each socioeconomic characteristic.

[c] Model 2 is controlled for each socioeconomic characteristic, prefecture-level characteristics (percentage of the older adult population, population density, and number of acute care hospital beds per population), and PCR tests per population.

[d] Model 3 is controlled for each socioeconomic characteristic (only for household crowding, smoking rate, and obesity rate), prefecture-level characteristics (percentage of the older adult population, population density, and number of acute care hospital beds per population), other

© 2021 Yoshikawa Y et al. *JAMA Network Open.*

socioeconomic characteristics (household income adjusted by regional price parities, Gini coefficient, the proportion of the population receiving public assistance, educational attainment, and unemployment rate), and PCR tests per population.

© 2021 Yoshikawa Y et al. *JAMA Network Open.*

eTable 6. Japanese COVID-19 Incidence Rate, Mortality Rate, Incidence Rate Ratio, and Mortality Rate Ratio by Prefectural Socioeconomic Characteristics, Further Adjusted for PCR Tests per Population, as of February 13, 2021 (continued)

		Incidence RR (95% CI)[a]				Mortality RR (95% CI)[a]		
Socioeconomic variable	Incidence rate per 100 000 (95% CI)	Model 1[b]	Model 2[c]	Model 3[d]	Mortality rate per 100 000 (95% CI)	Model 1[b]	Model 2[c]	Model 3[d]
Percentage of workers in retail industry								
1st quintile	483.6 (481.3-485.8)	1 [Reference]	1 [Reference]	NA	7.09 (6.83-7.37)	1 [Reference]	1 [Reference]	NA
2nd quintile	284.8 (282.9-286.6)	0.59 (0.58-0.59)	1.12 (1.11-1.14)	NA	4.80 (4.56-5.05)	0.68 (0.64-0.72)	1.34 (1.20-1.50)	NA
3rd quintile	314.9 (312.9-316.9)	0.65 (0.65-0.66)	1.17 (1.16-1.19)	NA	5.52 (5.25-5.79)	0.78 (0.73-0.83)	1.28 (1.18-1.39)	NA
4th quintile	118.2 (116.3-120.1)	0.24 (0.24-0.25)	0.90 (0.88-0.92)	NA	1.52 (1.32-1.76)	0.21 (0.18-0.25)	0.47 (0.40-0.56)	NA
5th quintile	222.9 (220.5-225.3)	0.46 (0.46-0.47)	1.39 (1.37-1.41)	NA	6.08 (5.70-6.49)	0.86 (0.80-0.92)	1.45 (1.31-1.61)	NA
Percentage of workers in transportation and postal industry								
1st quintile	114.4 (112.3-116.4)	1 [Reference]	1 [Reference]	NA	2.14 (1.87-2.44)	1 [Reference]	1 [Reference]	NA
2nd quintile	224.4 (221.9-226.9)	1.96 (1.92-2.00)	1.27 (1.24-1.30)	NA	3.72 (3.40-4.05)	1.73 (1.49-2.03)	1.40 (1.19-1.65)	NA
3rd quintile	108.5 (106.7-110.3)	0.95 (0.93-0.97)	0.80 (0.78-0.82)	NA	2.05 (1.82-2.31)	0.96 (0.80-1.14)	0.92 (0.77-1.11)	NA
4th quintile	423.8 (421.6-426.0)	3.71 (3.64-3.77)	1.30 (1.27-1.33)	NA	5.88 (5.62-6.14)	2.74 (2.40-3.15)	1.79 (1.54-2.08)	NA
5th quintile	385.3 (383.7-386.9)	3.37 (3.31-3.43)	1.61 (1.58-1.64)	NA	7.14 (6.91-7.36)	3.33 (2.92-3.82)	2.50 (2.18-2.90)	NA
Percentage of workers in restaurant industry								
1st quintile	76.3 (74.6-78.0)	1 [Reference]	1 [Reference]	NA	1.53 (1.30-1.79)	1 [Reference]	1 [Reference]	NA
2nd quintile	120.7 (119.0-122.4)	1.58 (1.54-1.62)	1.42 (1.39-1.46)	NA	2.42 (2.18-2.68)	1.58 (1.31-1.91)	1.56 (1.29-1.88)	NA
3rd quintile	151.7 (149.7-153.7)	1.99 (1.94-2.04)	1.59 (1.55-1.63)	NA	2.33 (2.08-2.59)	1.52 (1.26-1.84)	1.59 (1.31-1.93)	NA
4th quintile	277.1 (275.2-279.0)	3.63 (3.55-3.72)	2.69 (2.62-2.75)	NA	6.23 (5.95-6.53)	4.07 (3.47-4.81)	4.03 (3.41-4.79)	NA
5th quintile	496.6 (494.7-498.4)	6.51 (6.37-6.66)	2.49 (2.42-2.55)	NA	7.42 (7.20-7.65)	4.84 (4.14-5.71)	4.00 (3.33-4.83)	NA
Household crowding								
5th quintile	95.4 (93.8-97.1)	1 [Reference]	1 [Reference]	1 [Reference]	2.23 (1.99-2.49)	1 [Reference]	1 [Reference]	1 [Reference]
4th quintile	205.1 (202.8-207.4)	2.15 (2.11-2.19)	1.72 (1.68-1.76)	1.54 (1.51-1.57)	5.80 (5.42-6.20)	2.60 (2.29-2.97)	2.01 (1.75-2.30)	2.22 (1.93-2.57)
3rd quintile	149.4 (147.5-151.4)	1.57 (1.53-1.60)	1.40 (1.37-1.43)	1.29 (1.26-1.32)	2.79 (2.53-3.06)	1.25 (1.08-1.45)	1.13 (0.97-1.31)	1.03 (0.88-1.21)
2nd quintile	287.6 (285.8-289.5)	3.01 (2.96-3.07)	2.01 (1.97-2.05)	1.68 (1.64-1.71)	5.17 (4.92-5.42)	2.32 (2.06-2.62)	2.06 (1.81-2.35)	1.63 (1.41-1.88)
1st quintile	504.2 (502.3-506.2)	5.28 (5.19-5.38)	1.84 (1.80-1.87)	1.32 (1.28-1.35)	7.30 (7.06-7.53)	3.27 (2.92-3.68)	1.66 (1.45-1.92)	0.97 (0.81-1.17)
Smoking rate								
1st quintile	476.2 (473.7-478.7)	1 [Reference]	1 [Reference]	1 [Reference]	6.43 (6.14-6.72)	1 [Reference]	1 [Reference]	1 [Reference]
2nd quintile	275.4 (273.2-277.5)	0.58 (0.57-0.58)	1.05 (1.03-1.06)	1.28 (1.26-1.30)	4.27 (4.01-4.55)	0.66 (0.61-0.72)	1.08 (0.99-1.18)	1.08 (0.96-1.20)
3rd quintile	246.8 (244.9-248.7)	0.52 (0.51-0.52)	0.98 (0.97-0.99)	1.78 (1.75-1.82)	4.47 (4.22-4.73)	0.70 (0.65-0.75)	1.29 (1.17-1.42)	2.03 (1.74-2.37)
4th quintile	336.8 (334.4-339.2)	0.71 (0.70-0.71)	1.18 (1.17-1.19)	2.12 (2.06-2.17)	6.75 (6.41-7.10)	1.05 (0.98-1.12)	1.38 (1.29-1.49)	1.74 (1.46-2.06)
5th quintile	272.1 (270.1-274.2)	0.57 (0.57-0.58)	1.28 (1.26-1.29)	1.70 (1.67-1.73)	5.41 (5.12-5.70)	0.84 (0.78-0.90)	1.32 (1.21-1.45)	1.63 (1.41-1.90)
Obesity rate								
1st quintile	205.1 (203.1-207.2)	1 [Reference]	1 [Reference]	1 [Reference]	4.45 (4.16-4.76)	1 [Reference]	1 [Reference]	1 [Reference]
2nd quintile	467.1 (465.1-469.1)	2.28 (2.25-2.30)	0.85 (0.84-0.86)	0.94 (0.93-0.96)	7.10 (6.85-7.35)	1.59 (1.48-1.72)	0.83 (0.76-0.92)	0.91 (0.81-1.03)
3rd quintile	335.1 (333.2-337.1)	1.63 (1.62-1.65)	1.09 (1.08-1.10)	1.00 (0.99-1.02)	5.05 (4.81-5.30)	1.13 (1.05-1.23)	0.95 (0.87-1.03)	0.86 (0.78-0.95)
4th quintile	148.3 (146.2-150.4)	0.72 (0.71-0.74)	0.86 (0.84-0.87)	1.00 (0.97-1.02)	2.53 (2.27-2.83)	0.57 (0.50-0.65)	0.61 (0.54-0.70)	0.80 (0.69-0.93)
5th quintile	213.3 (211.1-215.5)	1.04 (1.03-1.05)	0.88 (0.87-0.90)	1.00 (0.98-1.02)	5.47 (5.12-5.84)	1.23 (1.12-1.35)	1.03 (0.93-1.14)	1.29 (1.12-1.49)

Abbreviations: NA, not applicable; RR, rate ratio.

[a] Incidence and mortality rate ratios were calculated using Poisson regression models with log(population) as the offset.

[b] Model 1 is controlled for each socioeconomic characteristic.

[c] Model 2 is controlled for each socioeconomic characteristic, prefecture-level characteristics (percentage of the older adult population, population density, and number of acute care hospital beds per population), and PCR tests per population.

[d] Model 3 is controlled for each socioeconomic characteristic (only for household crowding, smoking rate, and obesity rate), prefecture-level characteristics (percentage of the older adult population, population density, and number of acute care hospital beds per population), other

© 2021 Yoshikawa Y et al. *JAMA Network Open.*

socioeconomic characteristics (household income adjusted by regional price parities, Gini coefficient, the proportion of the population receiving public assistance, educational attainment, and unemployment rate), and PCR tests per population.

© 2021 Yoshikawa Y et al. *JAMA Network Open.*

eTable 7. Japanese COVID-19 Incidence Rate Ratio and Mortality Rate Ratio by Prefectural Socioeconomic Characteristics, with Sex- and Age-Adjusted by Indirect Standardization, as of February 13, 2021

		Incidence RR (95% CI)[a]				Mortality RR (95% CI)[a]		
Socioeconomic variable		Model 1[b]	Model 2[c]	Model 3[d]		Model 1[b]	Model 2[c]	Model 3[d]
Household income adjusted by regional price parities								
	5th quintile	1 [Reference]	1 [Reference]	NA		1 [Reference]	1 [Reference]	NA
	4th quintile	1.81 (1.79-1.82)	0.99 (0.98-1.00)	NA		1.45 (1.35-1.55)	0.77 (0.71-0.84)	NA
	3rd quintile	0.70 (0.69-0.71)	1.01 (1.00-1.03)	NA		0.83 (0.75-0.91)	1.16 (1.04-1.29)	NA
	2nd quintile	1.33 (1.32-1.35)	1.31 (1.29-1.33)	NA		1.49 (1.38-1.60)	1.37 (1.24-1.52)	NA
	1st quintile	0.96 (0.95-0.97)	1.46 (1.44-1.49)	NA		1.18 (1.08-1.28)	1.83 (1.60-2.09)	NA
Gini coefficient								
	1st quintile	1 [Reference]	1 [Reference]	NA		1 [Reference]	1 [Reference]	NA
	2nd quintile	1.07 (1.05-1.08)	1.05 (1.04-1.07)	NA		1.53 (1.39-1.69)	1.33 (1.19-1.47)	NA
	3rd quintile	1.69 (1.67-1.71)	0.96 (0.95-0.97)	NA		2.04 (1.87-2.23)	1.26 (1.15-1.38)	NA
	4th quintile	1.39 (1.37-1.41)	1.33 (1.31-1.36)	NA		1.93 (1.76-2.12)	1.40 (1.24-1.58)	NA
	5th quintile	2.54 (2.51-2.56)	0.82 (0.80-0.83)	NA		2.66 (2.45-2.90)	0.79 (0.69-0.91)	NA
Proportion of the population receiving public assistance								
	1st quintile	1 [Reference]	1 [Reference]	NA		1 [Reference]	1 [Reference]	NA
	2nd quintile	0.90 (0.88-0.91)	1.11 (1.10-1.13)	NA		1.00 (0.91-1.10)	1.11 (1.01-1.22)	NA
	3rd quintile	0.68 (0.67-0.69)	0.92 (0.90-0.93)	NA		0.52 (0.46-0.60)	0.63 (0.55-0.72)	NA
	4th quintile	1.69 (1.67-1.71)	1.54 (1.52-1.56)	NA		1.35 (1.24-1.47)	1.25 (1.15-1.36)	NA
	5th quintile	2.38 (2.36-2.41)	1.55 (1.52-1.57)	NA		2.15 (2.00-2.32)	1.56 (1.39-1.75)	NA
Educational attainment: college or higher-level degree								
	5th quintile	1 [Reference]	1 [Reference]	NA		1 [Reference]	1 [Reference]	NA
	4th quintile	0.40 (0.40-0.41)	0.58 (0.57-0.58)	NA		0.34 (0.31-0.38)	0.37 (0.34-0.41)	NA
	3rd quintile	0.44 (0.44-0.45)	0.71 (0.70-0.72)	NA		0.40 (0.37-0.44)	0.52 (0.47-0.58)	NA
	2nd quintile	0.47 (0.47-0.48)	0.78 (0.77-0.80)	NA		0.64 (0.60-0.69)	0.65 (0.58-0.73)	NA
	1st quintile	0.18 (0.18-0.18)	0.38 (0.37-0.39)	NA		0.18 (0.16-0.20)	0.25 (0.21-0.29)	NA
Unemployment rate								
	1st quintile	1 [Reference]	1 [Reference]	NA		1 [Reference]	1 [Reference]	NA
	2nd quintile	1.53 (1.50-1.55)	1.03 (1.01-1.05)	NA		1.40 (1.23-1.59)	1.08 (0.95-1.23)	NA
	3rd quintile	2.40 (2.36-2.45)	1.39 (1.36-1.41)	NA		1.95 (1.73-2.20)	1.31 (1.15-1.49)	NA
	4th quintile	3.51 (3.46-3.57)	1.39 (1.36-1.41)	NA		2.88 (2.58-3.23)	1.39 (1.23-1.58)	NA
	5th quintile	2.53 (2.49-2.57)	1.55 (1.52-1.58)	NA		2.85 (2.55-3.20)	1.90 (1.69-2.14)	NA
Percentage of workers in health care industry								
	1st quintile	1 [Reference]	1 [Reference]	NA		1 [Reference]	1 [Reference]	NA
	2nd quintile	0.33 (0.33-0.34)	0.69 (0.68-0.70)	NA		0.37 (0.33-0.41)	0.64 (0.57-0.71)	NA
	3rd quintile	0.77 (0.77-0.78)	1.28 (1.26-1.29)	NA		1.24 (1.17-1.30)	1.75 (1.60-1.90)	NA
	4th quintile	0.55 (0.55-0.56)	0.92 (0.90-0.93)	NA		0.46 (0.41-0.50)	0.61 (0.54-0.69)	NA
	5th quintile	0.29 (0.29-0.30)	0.79 (0.77-0.81)	NA		0.26 (0.23-0.30)	0.53 (0.45-0.64)	NA

Abbreviations: NA, not applicable; RR, rate ratio.

[a] Incidence and mortality rate ratios were calculated using Poisson regression models with log(expected cases or deaths) as the offset. Expected cases or deaths were estimated by indirect age and sex standardization.

[b] Model 1 is controlled for each socioeconomic characteristic.

[c] Model 2 is controlled for each socioeconomic characteristic, prefecture-level characteristics (percentage of the older adult population, population density, and number of acute care hospital beds per population), and PCR tests per population.

[d] Model 3 is controlled for each socioeconomic characteristic (only for household crowding, smoking rate, and obesity rate), prefecture-level characteristics (percentage of the older adult population, population density, and number of acute care hospital beds per population), other

© 2021 Yoshikawa Y et al. *JAMA Network Open.*

socioeconomic characteristics (household income adjusted by regional price parities, Gini coefficient, the proportion of the population receiving public assistance, educational attainment, and unemployment rate), and PCR tests per population.

© 2021 Yoshikawa Y et al. *JAMA Network Open.*

eTable 7. Japanese COVID-19 Incidence Rate Ratio and Mortality Rate Ratio by Prefectural Socioeconomic Characteristics, with Sex- and Age-Adjusted by Indirect Standardization, as of February 13, 2021 (continued)

Socioeconomic variable	Incidence RR (95% CI)[a]				Mortality RR (95% CI)[a]		
	Model 1[b]	Model 2[c]	Model 3[d]		Model 1[b]	Model 2[c]	Model 3[d]
Percentage of workers in retail industry							
1st quintile	1 [Reference]	1 [Reference]	NA		1 [Reference]	1 [Reference]	NA
2nd quintile	0.60 (0.59-0.60)	1.24 (1.22-1.26)	NA		0.67 (0.63-0.71)	1.48 (1.33-1.64)	NA
3rd quintile	0.66 (0.65-0.67)	1.18 (1.16-1.19)	NA		0.75 (0.71-0.80)	1.32 (1.22-1.43)	NA
4th quintile	0.25 (0.25-0.26)	0.76 (0.75-0.78)	NA		0.18 (0.15-0.21)	0.43 (0.37-0.51)	NA
5th quintile	0.48 (0.47-0.48)	1.33 (1.31-1.35)	NA		0.71 (0.66-0.77)	1.47 (1.33-1.63)	NA
Percentage of workers in transportation and postal industry							
1st quintile	1 [Reference]	1 [Reference]	NA		1 [Reference]	1 [Reference]	NA
2nd quintile	1.93 (1.89-1.97)	1.27 (1.25-1.30)	NA		1.94 (1.66-2.27)	1.41 (1.20-1.66)	NA
3rd quintile	0.94 (0.91-0.96)	0.77 (0.76-0.79)	NA		1.04 (0.87-1.24)	0.92 (0.77-1.10)	NA
4th quintile	3.55 (3.49-3.62)	1.28 (1.26-1.31)	NA		3.41 (2.98-3.92)	1.83 (1.58-2.14)	NA
5th quintile	3.22 (3.16-3.28)	1.59 (1.56-1.62)	NA		4.25 (3.72-4.87)	2.68 (2.32-3.09)	NA
Percentage of workers in restaurant industry							
1st quintile	1 [Reference]	1 [Reference]	NA		1 [Reference]	1 [Reference]	NA
2nd quintile	1.57 (1.53-1.61)	1.44 (1.40-1.48)	NA		1.65 (1.37-1.99)	1.57 (1.31-1.90)	NA
3rd quintile	1.96 (1.91-2.01)	1.55 (1.51-1.59)	NA		1.71 (1.42-2.08)	1.60 (1.32-1.95)	NA
4th quintile	3.54 (3.46-3.62)	2.78 (2.72-2.85)	NA		4.67 (3.98-5.53)	4.37 (3.70-5.19)	NA
5th quintile	6.14 (6.00-6.28)	2.58 (2.51-2.65)	NA		6.48 (5.55-7.64)	4.44 (3.70-5.36)	NA
Household crowding							
5th quintile	1 [Reference]	1 [Reference]	1 [Reference]		1 [Reference]	1 [Reference]	1 [Reference]
4th quintile	2.12 (2.08-2.17)	1.79 (1.76-1.83)	1.50 (1.47-1.53)		2.74 (2.41-3.12)	2.20 (1.93-2.52)	2.26 (1.96-2.62)
3rd quintile	1.55 (1.52-1.59)	1.29 (1.26-1.32)	1.22 (1.20-1.25)		1.33 (1.15-1.54)	1.07 (0.92-1.25)	1.01 (0.87-1.18)
2nd quintile	2.93 (2.88-2.98)	1.98 (1.94-2.02)	1.65 (1.62-1.68)		2.78 (2.46-3.14)	2.10 (1.85-2.40)	1.68 (1.46-1.93)
1st quintile	5.02 (4.93-5.11)	2.03 (1.99-2.07)	1.35 (1.32-1.39)		4.19 (3.74-4.72)	1.90 (1.65-2.18)	1.05 (0.89-1.26)
Smoking rate							
1st quintile	1 [Reference]	1 [Reference]	1 [Reference]		1 [Reference]	1 [Reference]	1 [Reference]
2nd quintile	0.59 (0.58-0.59)	1.01 (0.99-1.02)	1.25 (1.23-1.27)		0.62 (0.58-0.67)	1.05 (0.97-1.15)	1.06 (0.95-1.18)
3rd quintile	0.53 (0.53-0.53)	1.00 (0.99-1.02)	1.78 (1.74-1.82)		0.67 (0.62-0.72)	1.34 (1.22-1.47)	2.05 (1.77-2.39)
4th quintile	0.72 (0.72-0.73)	1.19 (1.18-1.21)	2.04 (1.99-2.10)		1.00 (0.94-1.07)	1.43 (1.33-1.53)	1.72 (1.46-2.04)
5th quintile	0.58 (0.58-0.59)	1.36 (1.35-1.38)	1.61 (1.58-1.64)		0.77 (0.72-0.82)	1.49 (1.37-1.63)	1.58 (1.37-1.83)
Obesity rate							
1st quintile	1 [Reference]	1 [Reference]	1 [Reference]		1 [Reference]	1 [Reference]	1 [Reference]
2nd quintile	2.20 (2.18-2.23)	0.87 (0.85-0.88)	0.92 (0.90-0.94)		1.87 (1.73-2.02)	0.86 (0.78-0.95)	0.90 (0.79-1.01)
3rd quintile	1.60 (1.58-1.62)	1.12 (1.11-1.13)	0.99 (0.97-1.00)		1.28 (1.18-1.39)	0.99 (0.90-1.08)	0.86 (0.78-0.94)
4th quintile	0.73 (0.72-0.74)	0.79 (0.78-0.81)	0.91 (0.89-0.93)		0.55 (0.49-0.63)	0.56 (0.49-0.63)	0.71 (0.61-0.82)
5th quintile	1.04 (1.02-1.05)	0.89 (0.87-0.90)	0.92 (0.90-0.94)		1.20 (1.09-1.32)	1.03 (0.93-1.14)	1.15 (1.00-1.33)

Abbreviations: NA, not applicable; RR, rate ratio.

[a] Incidence and mortality rate ratios were calculated using Poisson regression models with log(expected cases or deaths) as the offset. Expected cases or deaths were estimated by indirect age and sex standardization.

[b] Model 1 is controlled for each socioeconomic characteristic.

[c] Model 2 is controlled for each socioeconomic characteristic, prefecture-level characteristics (percentage of the older adult population, population density, and number of acute care hospital beds per population), and PCR tests per population.

[d] Model 3 is controlled for each socioeconomic characteristic (only for household crowding, smoking rate, and obesity rate), prefecture-level characteristics (percentage of the older adult population, population density, and number of acute care hospital beds per population), other

© 2021 Yoshikawa Y et al. *JAMA Network Open.*

socioeconomic characteristics (household income adjusted by regional price parities, Gini coefficient, the proportion of the population receiving public assistance, educational attainment, and unemployment rate), and PCR tests per population.

© 2021 Yoshikawa Y et al. *JAMA Network Open.*

eReferences

1. Statistics Bureau, Ministry of Internal Affairs and Communications, Japan. National Survey of Family Income and Expenditure. Accessed February 1, 2021. http://www.stat.go.jp/english/data/zensho/index.html

2. Statistics Bureau, Ministry of Internal Affairs and Communications, Japan. Results of Retail Price Survey (Structural Survey). Accessed February 1, 2021. http://www.stat.go.jp/english/data/kouri/kouzou/k_kekka.html#i1

3. Ministry of Health, Labour and Welfare, Japan. Results of National Survey on Public Assistance Recipients. Accessed February 1, 2021. https://www.mhlw.go.jp/toukei/list/74-16.html

4. Statistics Bureau, Ministry of Internal Affairs and Communications, Japan. Employment Status Survey. Accessed February 1, 2021. https://www.stat.go.jp/data/shugyou/2017/index.html

5. Statistics Bureau, Ministry of Internal Affairs and Communications, Japan. Labour Force Survey. Accessed February 1, 2021. https://www.stat.go.jp/english/data/roudou/index.html

6. Statistics Bureau, Ministry of Internal Affairs and Communications, Japan. Housing and Land Survey. Accessed February 1, 2021. http://www.stat.go.jp/data/jyutaku/index.html

7. Ministry of Health, Labour and Welfare, Japan. Results of Comprehensive Survey of Living Conditions. Accessed February 1, 2021. https://www.mhlw.go.jp/toukei/saikin/hw/k-tyosa/k-tyosa19/index.html

8. Ministry of Health, Labour and Welfare, Japan. 5th NDB Open Data Japan. Accessed February 1, 2021. https://www.mhlw.go.jp/stf/seisakunitsuite/bunya/0000177221_00008.html

9. Statistics Bureau, Ministry of Internal Affairs and Communications, Japan. Result of the Population Estimates. Accessed February 1, 2021. https://www.stat.go.jp/english/data/jinsui/2.html

10. Statistics Bureau, Ministry of Internal Affairs and Communications, Japan. 2015 Population Census: Summary of the results and statistical tables. Accessed February 1, 2021. https://www.stat.go.jp/english/data/kokusei/2015/summary.html

11. Ministry of Health, Labour and Welfare, Japan. Hospital Bed Function Report 2018. Accessed February 1, 2021. https://www.mhlw.go.jp/stf/seisakunitsuite/bunya/open_data_00005.html

12. Ministry of Health, Labour and Welfare, Japan. COVID-19 Updates in Japan. Accessed February 14, 2021. https://www.mhlw.go.jp/stf/covid-19/kokunainohasseijoukyou.html

© 2021 Yoshikawa Y et al. *JAMA Network Open.*
